# Unveiling the Microbial Community of Naturally Fermented Bella di Cerignola PDO Table Olives Integrating Culture-Dependent, MALDI-TOF MS Identification and Long-Read Metabarcoding Approaches

**DOI:** 10.3390/foods15203685

**Published:** 2026-10-16

**Authors:** Katia Gialluisi, Giuseppe Petruzzino, Rosa Anna Siciliano, Maria Fiorella Mazzeo, Vittorio Capozzi, Giancarlo Perrone, Luciana De Vero, Massimo Ferrara

**Affiliations:** 1 Institute of Sciences of Food Production (ISPA), National Research Council of Italy (CNR), Via Giovanni Amendola 122/O, 70126 Bari, Italy; katia.gialluisi@cnr.it (K.G.); giuseppe.petruzzino@cnr.it (G.P.); giancarlo.perrone@cnr.it (G.P.); massimo.ferrara@cnr.it (M.F.); 2 Institute of Food Sciences (ISA), National Research Council of Italy (CNR), Via Roma 64, 83100 Avellino, Italy; rosa.siciliano@isa.cnr.it (R.A.S.); fiorella.mazzeo@isa.cnr.it (M.F.M.); 3 Institute of Sciences of Food Production (ISPA), National Research Council of Italy (CNR), c/o CS-DAT, Via Michele Protano, 71121 Foggia, Italy; vittorio.capozzi@cnr.it

**Keywords:** spontaneous fermentation, olive microbiota, lactic acid bacteria, yeasts, MALDI-TOF MS, metabarcoding analysis

## Abstract

Spontaneous fermentation of table olives is driven by complex microbial communities that influence product quality and safety. This study investigated the microbial community during the intermediate and late stages (from 3 to 6 months) of spontaneous fermentation of *Bella di Cerignola* table olives in brine using an integrated culture-dependent and culture-independent approach. Bacterial and fungal communities were monitored using viable counts, isolation and identification by MALDI-TOF MS, and long-read metabarcoding targeting the 16S rRNA gene and ITS region. Lactic acid bacteria remained dominant throughout fermentation, whereas the yeast population progressively declined during the last period. MALDI-TOF MS identified *Lactiplantibacillus pentosus* as the predominant culturable bacterial species and *Pichia membranifaciens*, *Pichia manshurica*, and *Wickerhamomyces anomalus* as the dominant yeasts. Metabarcoding confirmed the ecological relevance of these yeasts and revealed a bacterial community dominated by the *Secundilactobacillus collinoides*/*paracollinoides* group, which was not recovered by cultivation, and the *Lactiplantibacillus plantarum* group. The integration of MALDI-TOF MS and long-read metabarcoding analysis provided a comprehensive overview of the microbial community of *Bella di Cerignola* olives, demonstrating the complementary value of the two approaches for the microbiota characterisation of spontaneous olive fermentations and for the identification of autochthonous candidate strains to further investigate as starter cultures.

## 1. Introduction

Table olives represent one of the most widely consumed fermented vegetable products, particularly in Mediterranean countries, both for their economic impact and their nutritional value. In most traditional productions, table olive fermentation occurs spontaneously without the addition of starter cultures, relying instead on the indigenous microbiota naturally present on the fruit surface and in the processing environment [1]. Several intrinsic and extrinsic factors, including cultivar, fruit maturity, salt concentration, temperature, pH, and water activity, strongly influence the dynamics of these microbial populations. Therefore, spontaneous fermentations are characterised by a clear microbial succession, where an initially heterogeneous microbiota evolves toward a more stable ecosystem dominated by adapted species that shape the final product quality [1,2].

Among the different cultivars, *Bella di Cerignola* is one of the most important Italian table olive varieties, widely cultivated in the Apulia region and recognised with the related *La Bella della Daunia* Protected Designation of Origin (PDO) status. This cultivar is worldwide renowned for its impressive size, firm flesh, and high organoleptic quality, typically processed through natural fermentation in brine to preserve its distinctive sensory attributes [3]. This consortium is primarily composed of lactic acid bacteria (LAB) and yeasts [4]. The initial stages of fermentation are dominated by LAB species such as *Lactiplantibacillus plantarum* and *Lactiplantibacillus pentosus*, which play a key role in the acidification of the brine, ensuring the microbiological safety and shelf-life of the product. Concurrently, yeasts (e.g., *Pichia*, *Wickerhamomyces*, and *Candida* spp.) contribute to the development of the aromatic profile and the degradation of oleuropein, the compound responsible for the olive’s natural bitterness [2,5].

Traditionally, the study of olive microbiota has relied on culture-dependent techniques, including plate counts and phenotypic or biochemical identification of isolates. While these approaches provide valuable information on viable and technologically relevant microorganisms, they capture only a fraction of the total microbial diversity present in fermented foods. In this context, the introduction of rapid and accurate identification tools such as Matrix-Assisted Laser Desorption Ionization Time-of-Flight Mass Spectrometry (MALDI-TOF MS) has significantly improved the resolution and efficiency of microbial characterisation, allowing high-throughput and cost-effective identification of isolates [6,7].

More recently, culture-independent approaches based on next-generation sequencing (NGS), including metagenomic and metabarcoding, have revolutionised the study of food microbial ecosystems. These techniques enable comprehensive profiling of microbial communities, including non-culturable microorganisms, and provide deeper insights into microbial diversity, structure, and potential functionality. The integration of culture-dependent and culture-independent methods is therefore increasingly recognised as a powerful strategy to obtain a more complete and accurate understanding of the microbial community involved in fermentation processes [8,9].

Despite growing interest in olive microbiology, detailed studies on the temporal evolution of microbial communities during spontaneous fermentation of specific cultivars, such as *Bella di Cerignola* (a cultivar with high economic relevance in the Mediterranean table olive sector), remain limited, particularly when integrating traditional microbiological techniques with advanced analytical approaches. Previous investigations have identified *Lactiplantibacillus* spp. and yeast species belonging to *Wickerhamomyces anomalus* and *Pichia* spp. [10,11] as important members of the fermentation microbiota, but the temporal persistence of these microorganisms and the contribution of non-culturable taxa remain poorly understood. Moreover, the limited taxonomic resolution of conventional short-read metabarcoding approaches often hampers discrimination among closely related bacterial or yeast species. The recent availability of long-read sequencing platforms overcomes some of these limitations, offering improved taxonomic resolution compared to conventional approaches and enabling more accurate characterisation of microbial communities at the species level. Consequently, integrating cultivation-based techniques with long-read metabarcoding may provide novel insights into the microbial community during spontaneous olive fermentation. Indeed, understanding microbial succession during fermentation is essential because changes in community composition directly influence acidification dynamics, product safety, sensory properties, and final quality.

We hypothesised that the integration of cultivation-based analyses with long-read metabarcoding would reveal a broader microbial diversity than either approach alone and would provide a more comprehensive description of the microbial community during fermentation. Then, our study investigated the microbial community occurring during the intermediate and late stages of spontaneous fermentation of *Bella di Cerignola* table olives in brine. Particular attention was devoted to comparing culture-dependent and culture-independent methodologies to identify the core microbiota driving fermentation and to evaluate the occurrence of potentially relevant taxa that may remain undetected by traditional isolation procedures. To this end, samples collected at 3, 4, and 6 months of fermentation were analysed through an integrated culture-dependent and culture-independent approach, comprising microbial enumeration, isolation and MALDI-TOF MS identification of representative colonies, and long-read metabarcoding. By integrating these complementary methodologies, our study provides novel insights into the olive-associated microbiota during the intermediate and late phases of fermentation. These findings enhance our understanding of the microbial community involved in the process and may support future optimisation strategies, including the selection of autochthonous candidate strains to be used as starter cultures.

## 2. Materials and Methods

### 2.1. Sampling

The table olive samples used in this study consisted of *Bella di Cerignola cultivar*/*La Bella della Daunia* PDO harvested in November 2025 in the Apulia region. The samples were provided by the Consorzio “La Bella della Daunia D.O.P” (Cerignola, Apulia, Italy), where they are processed according to the traditional natural (also named Greek-style) method (spontaneous fermentation), allowing the development of autochthonous microbial communities naturally present on the olive fruit and in the processing environment. In this natural-style processing method, debittering is achieved through slow spontaneous fermentation in brine, without an alkaline treatment. Fermentation is initiated at a NaCl concentration of approximately 10%; during the first days, salinity decreases rapidly because of osmotic exchanges between the fruits and the brine and subsequently tends to stabilise, reaching a final value of approximately 7%. At the end of the fermentation process, the total acidity, expressed as citric acid monohydrate, is approximately 0.22%, and the pH remains stably below 4.5.

In our work, the fermentation process was monitored from the third to the sixth month, thus covering the intermediate and final stages of the fermentation period, which typically lasts about six months for the spontaneous fermentation of naturally processed table olives. The samples, supplied by the Consorzio in three 5 L plastic containers filled with olives in brine, originated from the same vessel of an industrial production batch. The sampling was performed at three different time points, corresponding to 3 (M3), 4 (M4) and 6 (M6) months of spontaneous fermentation, to evaluate the temporal evolution of microbial communities during the process. Olive and brine samples were processed immediately after collection. The pH of the brine was measured at each sampling point using a calibrated digital pH meter (Orion, Thermo Fisher Scientific, Waltham, MA, USA) to monitor acidification during the fermentation.

### 2.2. Sample Preparation and Microbiological Analysis

For microbiological analyses, at each sampling time, three olive samples were collected (one from each container) and subjected to the subsequent microbiological and molecular analyses, according to standard operating procedures (SOPs) for fermented foods [12] with minor modifications. Ten grams of olives were weighed and homogenised with 90 mL of the corresponding brine using a Stomacher laboratory blender (Seward Ltd., Worthing, UK) for 2 min. The homogenate was subjected to low-speed centrifugation (4000 rpm, 5 min, 4 °C) to remove coarse solid particles, and the resulting supernatant was transferred to sterile tubes and centrifuged again (13,000 rpm, 20 min, 4 °C) to collect microbial biomass. This procedure was adopted to obtain a more homogeneous microbial suspension and was applied consistently to all samples and biological replicates. The pellet obtained was washed with 30 mL of sterile physiological saline solution (0.9% NaCl), followed by a further centrifugation step at 13,000 rpm for 10 min. After washing, the pellet was resuspended in 30 mL of sterile physiological saline solution and serial decimal dilutions were prepared from the suspension for microbiological enumeration on different selective media. Each sample consisted of three independent biological replicates, and each replicate was processed separately throughout all analytical steps, from sample preparation to plating.

The fungi population was enumerated on Dichloran Rose Bengal Agar plates (DRBC, Biolife, Milan, Italy) supplemented with 0.1 g/L chloramphenicol and incubated at 25 °C for 48 h. Regarding bacterial population, De Man-Rogosa-Sharpe agar (MRS, Oxoid Ltd., Basingstoke, UK) and M17 agar (Liofilchem, Roseto degli Abruzzi, Italy) plates, both supplemented with 0.05 g/L cycloheximide to inhibit fungal growth, were used for the enumeration of lactic acid bacteria (LAB). Both media were incubated at 30 °C for 48 h.

After incubation, colonies were counted, and the results were expressed as log10 colony-forming units per mL (log CFU/mL).

### 2.3. Culturable Microbiota Identified by MALDI-TOF MS

At each fermentation time point (M3, M4 and M6), representative colonies were isolated after the incubation period in order to characterise the culturable microbial diversity. Approximately 10 to 20 colonies of yeasts and bacteria were randomly selected based on morphological differences (e.g., colony size, shape, and colour) to ensure adequate representation of the microbial populations. Yeast isolates were streaked onto Potato Dextrose Agar (PDA, Oxoid Ltd., Basingstoke, UK), while bacterial isolates, mainly consisting of LAB, were streaked onto MRS agar. Purification was performed through three successive streaking steps to obtain pure cultures of the yeasts and bacterial isolates. Their identification at species level was carried out by MALDI-TOF MS using a MALDI Biotyper Sirius instrument (Bruker Daltonics, Bremen, Germany). For sample preparation, colonies were deposited in duplicate on an MSP 96 polished steel (Bruker Daltonics, Bremen, Germany) target and treated with 1 μL of 70% formic acid. After drying at 35 °C in the MBT FAST Shuttle device (Bruker Daltonics, Bremen, Germany), 1 μL of HCCA matrix solution (10 mg/mL α-cyano-4-hydroxycinnamic acid dissolved in 50% acetonitrile, 47.5% HPLC-grade water, and 2.5% trifluoroacetic acid) was applied to each spot and allowed to dry at the same temperature. Instrument calibration was carried out with the Bruker Bacterial Test Standard (BTS). Mass spectra were automatically acquired in positive linear mode across a mass range of 2000–20,000 *m*/*z* using the FlexControl software v3.4 and the MBT_Auto_X method. The resulting protein fingerprints were analysed with MBT Compass HT v5.1.410 software, and species-level identifications were achieved by comparing acquired mass spectra with those present in the MBT Compass Library (reference database), which includes 12,600 main spectral profiles representing approximately 700 genera and 4300 species. According to the manufacturer’s recommendations, score values of 2.00 or higher were accepted as high-confidence species-level identifications.

### 2.4. Long-Read Metabarcoding Analysis

Total microbial DNA was extracted from the same concentrated microbial suspension used for the culture-dependent analyses. Briefly, a 2 mL aliquot of each sample was centrifuged (13,000 rpm, 10 min, 4 °C) to pellet the microbial cells and the resulting pellet was subjected to DNA extraction using the QIAamp PowerFecal Pro DNA Kit (Qiagen, Hilden, Germany) following the manufacturer’s instructions. The concentration and purity of the extracted DNA were determined using a NanoDrop OneC spectrophotometer (Thermo Fisher Scientific, Waltham, MA, USA) and DNA integrity was further assessed by electrophoresis on a 0.8% (*w*/*v*) agarose gel stained with GelRed (Biotium, Fremont, CA, USA). The extracted DNA was used for metabarcoding analysis to characterise bacterial and fungal communities, using the kit Microbial Amplicon Barcoding Sequencing for 16S and ITS (SQK-MAB114.24, Oxford Nanopore Technologies, Oxford, UK), following the manufacturer’s instructions. The 18 barcoded libraries were pooled and sequenced on an R10.4.1 flow cell (FLO-MIN114) with a MinION Mk1D device (Oxford Nanopore Technologies, Oxford, UK) controlled by MinKNOW v25.09.16 [13], with real-time super-accurate basecalling (Dorado v7.11.2, model dna_r10.4.1_e8.2_400bps_sup@v5.2.0), a minimum mean quality score of 10 and barcode trimming. Quality-filtered reads were primer-trimmed with Cutadapt v5.2 [14]. Taxonomic classification [15] was performed with Emu v3.6.2 with default settings, using the Emu default database (rrnDB v5.6 and NCBI 16S RefSeq [16,17,18] for the 16S reads, and the Emu database derived from UNITE 2024 [19] for the ITS reads. No further trimming or length filtering was applied before classification. Species whose estimated abundance corresponds to fewer than ten reads were discarded; relative abundances were calculated as the proportions of the classified reads of each sample assigned to each species, genus-level abundances were obtained by summing the species-level values, and rarefaction was applied.

For comparison, the ITS reads were classified with dnabarcoder v1.0.7 [20] against the UNITE 2024 reference [19], and all reads, including 16S and ITS data output, were classified with Kraken2 v2.17.1 [21] against the PlusPFP-16 database. Alpha diversity (Shannon and Simpson indices and species richness, counted as the species with a relative abundance ≥ 0.01%) and beta diversity (Bray–Curtis dissimilarity, visualised by PCoA) were calculated from the species-level relative abundances with the vegan package in R. Alpha-diversity indices were compared among sampling times with the same repeated-measures ANOVA (*p* < 0.05) and Tukey’s HSD test (*p* < 0.05), and community composition by permutational multivariate analysis of variance (PERMANOVA) on Bray–Curtis dissimilarities, with all permutations of sampling times within containers.

### 2.5. Statistical Analysis

Statistical analyses were performed using R software (version 4.5.3). Viable counts (log CFU/mL) are expressed as mean ± standard deviation of three biological replicates. Differences among sampling times were analysed separately for each culture medium using a one-way repeated-measures analysis of variance (ANOVA), with time as the within-subject factor. When significant effects were detected, pairwise comparisons were performed using Tukey’s HSD post hoc test to account for multiple comparisons. In addition, *p*-values obtained from the three ANOVAs (one for each culture medium) were adjusted using the Holm correction method. Statistical significance was set at *p* ≤ 0.05.

## 3. Results

### 3.1. Evolution of Viable Microbial Communities During Spontaneous Fermentation of Bella di Cerignola Olives

**Figure 1:**
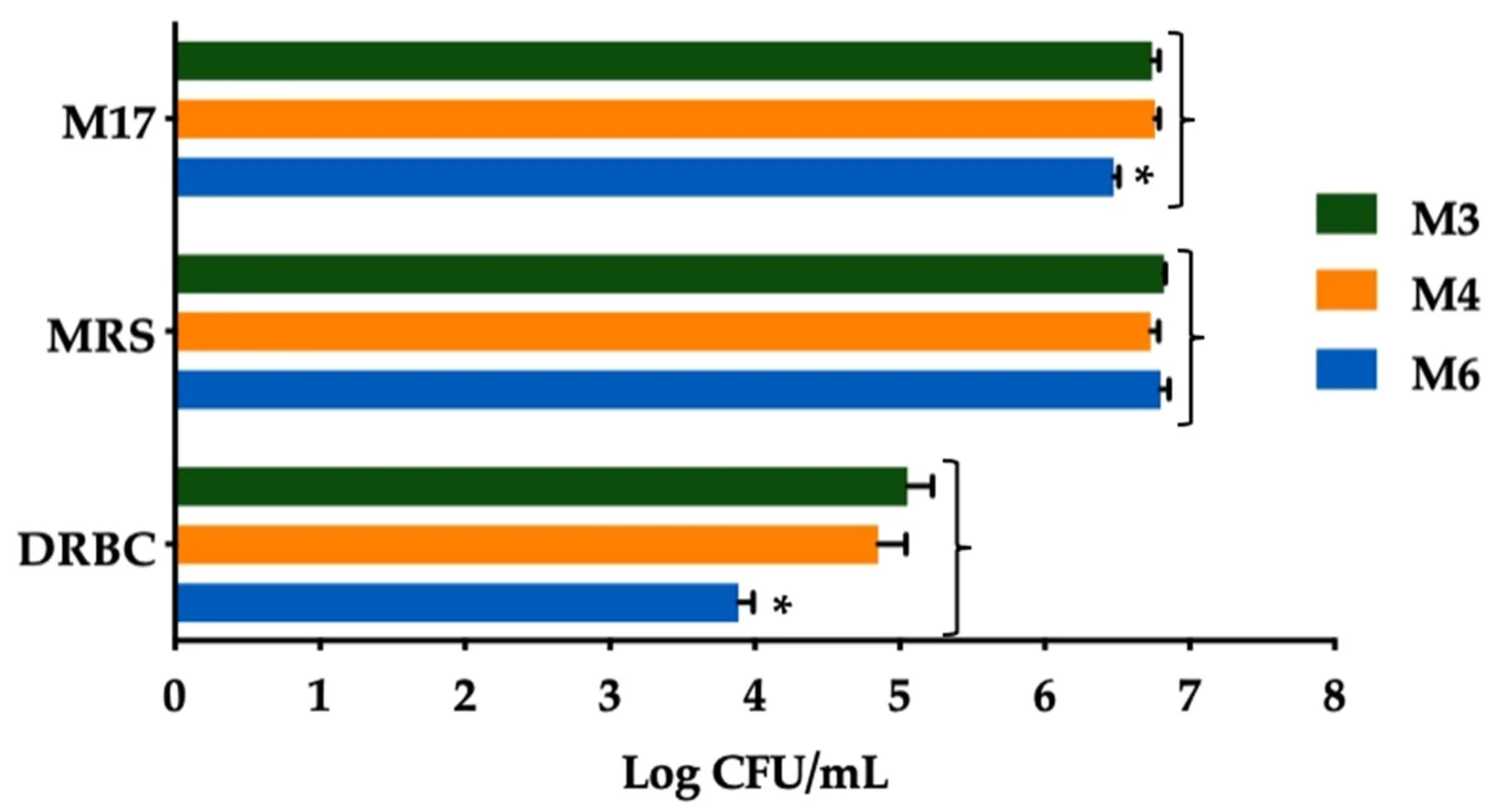
Viable counts (log CFU/mL) of bacteria (M17 and MRS) and yeasts (DRBC) during spontaneous fermentation of *Bella di Cerignola* olives at 3 (M3), 4 (M4), and 6 (M6) months. Bars represent the mean of three biological replicates, and error bars represent the standard deviation. Within each medium, bars with an asterisk (*) are significantly different (one-way repeated-measures ANOVA followed by Tukey’s HSD test, *p* ≤ 0.05). Curly brackets indicate the statistical comparison groups.

The evolution of the main microbial groups was evaluated at 3, 4, and 6 months of fermentation based on viable cell counts (log CFU/mL) on selective media (DRBC, MRS, and M17), as shown in Figure 1. The fermentation was carried out under spontaneous conditions, without the addition of starter cultures.

At M3, the microbial community was already well established, with LAB representing the dominant fraction. Cell densities reached 6.82 log CFU/mL on MRS and 6.74 log CFU/mL on M17, whereas yeasts (DRBC) were present at lower levels (5.05 log CFU/mL). At M4, microbial populations remained relatively stable, with LAB showing only minor variations (6.73 log CFU/mL on MRS and 6.76 log CFU/mL on M17), while yeast counts slightly decreased to 4.85 log CFU/mL.

At M6, a clearer shift in microbial composition was observed. LAB maintained high cell densities (6.80 log CFU/mL on MRS and 6.47 log CFU/mL on M17). The most pronounced change was observed for yeasts, whose counts declined to 3.89 log CFU/mL, corresponding to a reduction of more than 1 logarithmic unit compared with M3.

According to the repeated-measures ANOVA, fermentation time had a significant effect on yeast counts on DRBC (F(2, 4) = 45.35, *p* = 0.002) and on bacterial counts on M17 (F(2, 4) = 33.96, *p* = 0.003), and both effects remained significant after Holm correction for DRBC and M17 (*p* = 0.005 and *p* = 0.006, respectively). In both cases, counts at M6 were significantly lower than at M3 and M4 (Tukey’s HSD test, *p* < 0.01), whereas M3 and M4 did not differ significantly (*p* = 0.37 and *p* = 0.90, respectively). In contrast, counts on MRS did not change significantly during the monitored period (F(2, 4) = 4.64, *p* = 0.091), and the result remained non-significant after Holm correction (*p* = 0.091).

The pH values showed only minor variations throughout the investigated period, decreasing slightly from 4.064 ± 0.015 at M3 to 4.013 ± 0.008 at M4 and 3.976 ± 0.009 at M6. These results indicate that the fermentation environment remained consistently acidic during the monitored stages, with no substantial pH changes occurring between 3 and 6 months of fermentation.

### 3.2. Identification of Culturable Microorganisms by MALDI-TOF MS

**Figure 2:**
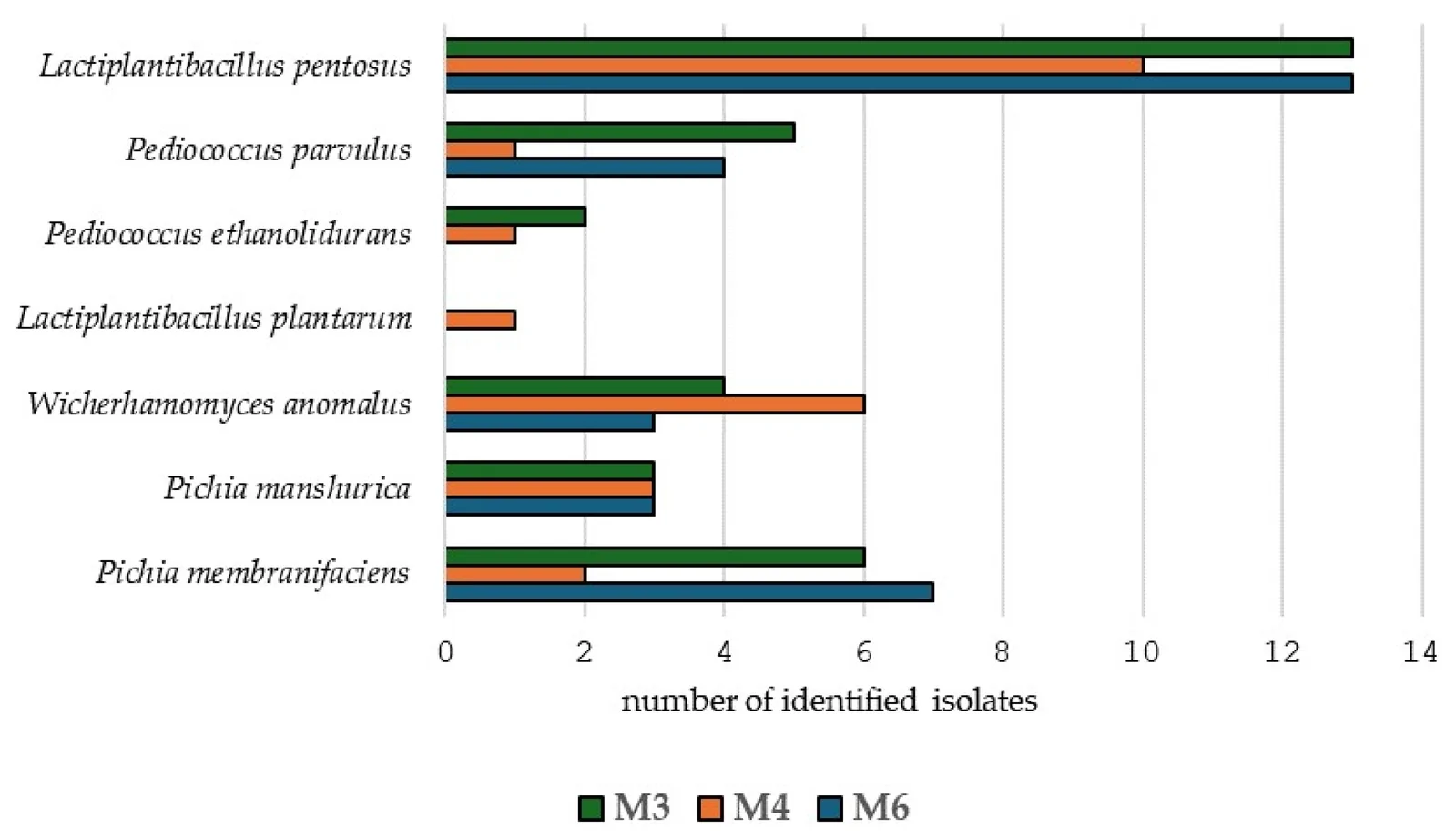
Yeast and bacterial isolates identified by MALDI-TOF MS at 3 (M3), 4 (M4), and 6 (M6) months of spontaneous fermentation of *Bella di Cerignola* olives. Values represent the number of isolates of each species identified at each sampling time.

A total of 87 isolates recovered from MRS, M17, and DRBC media at the three fermentation stages (M3, M4, and M6) were identified by MALDI-TOF MS (Table S1), and the results are summarised in Figure 2.

At M3, the bacterial community was dominated by *Lactiplantibacillus pentosus*, which accounted for all isolates recovered on MRS and for a substantial proportion of those isolated on M17. In addition, *Pediococcus parvulus* and *Pediococcus ethanolidurans* were identified. The yeast community was mainly represented by *Pichia membranifaciens*, *Pichia manshurica*, and *Wickerhamomyces anomalus*.

At M4, *L. pentosus* remained the predominant bacterial species, whereas the yeast community showed limited variation and was still composed of *P. manshurica*, *P. membranifaciens*, and *W. anomalus*. Only one isolate was identified as *Lactiplantibacillus plantarum*. At M6, *L. pentosus* continued to dominate the bacterial population, followed by *P. parvulus*. The yeast community remained relatively stable and was again represented by *P. membranifaciens*, *P. manshurica*, and *W. anomalus*.

### 3.3. Bacterial and Fungal Community Revealed by Long-Read Metabarcoding Analysis

To obtain a comprehensive characterisation of the microbiota associated with the spontaneous fermentation of *Bella di Cerignola* olives, total DNA was subjected to long-read metabarcoding analysis. The 22 h run produced 9.34 million reads, of which 8.81 million (94.4%; 10.07 Gb; read N50 1.51 kb) passed the quality filter; the number of reads analysed for each sample is reported in Table S2.

The bacterial community associated with the spontaneous fermentation of *Bella di Cerignola* olives was characterised by long-read 16S metabarcoding at M3, M4, and M6 (Table S3).

**Figure 3:**
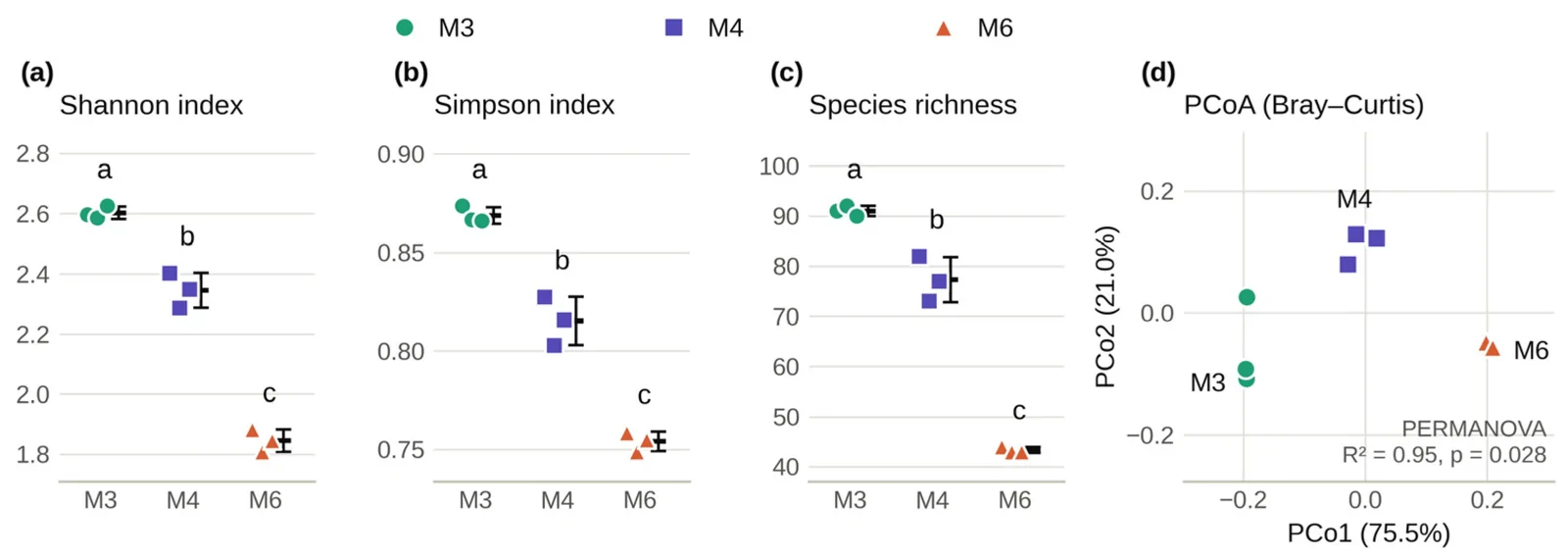
Alpha and beta diversity of the bacterial communities during spontaneous fermentation of *Bella di Cerignola* olives at 3 (M3), 4 (M4) and 6 (M6) months. Shannon index, Simpson index and species richness: each symbol is one biological replicate; the horizontal dash and the vertical bar indicate the mean and the standard deviation. Within each panel, time points with different letters are significantly different (repeated-measures ANOVA followed by Tukey’s HSD test, *p* ≤ 0.05) (**a**–**c**). Principal coordinate analysis (PCoA) of Bray–Curtis dissimilarities; each symbol is one sample, and the PERMANOVA result is given in each panel (**d**).

Bacterial alpha diversity decreased from M3 to M6 for all indices (Figure 3a–c; Shannon index 2.60 ± 0.02, 2.35 ± 0.06 and 1.85 ± 0.04; repeated-measures ANOVA, *p* < 0.001; Tukey’s HSD test, *p* < 0.01 for all pairwise comparisons). The PCoA analysis, based on Bray–Curtis dissimilarities (Figure 3d), indicated samples grouped by time point, and bacterial community composition differed among time points (PERMANOVA, R^2^ = 0.95, *p* = 0.028).

**Figure 4:**
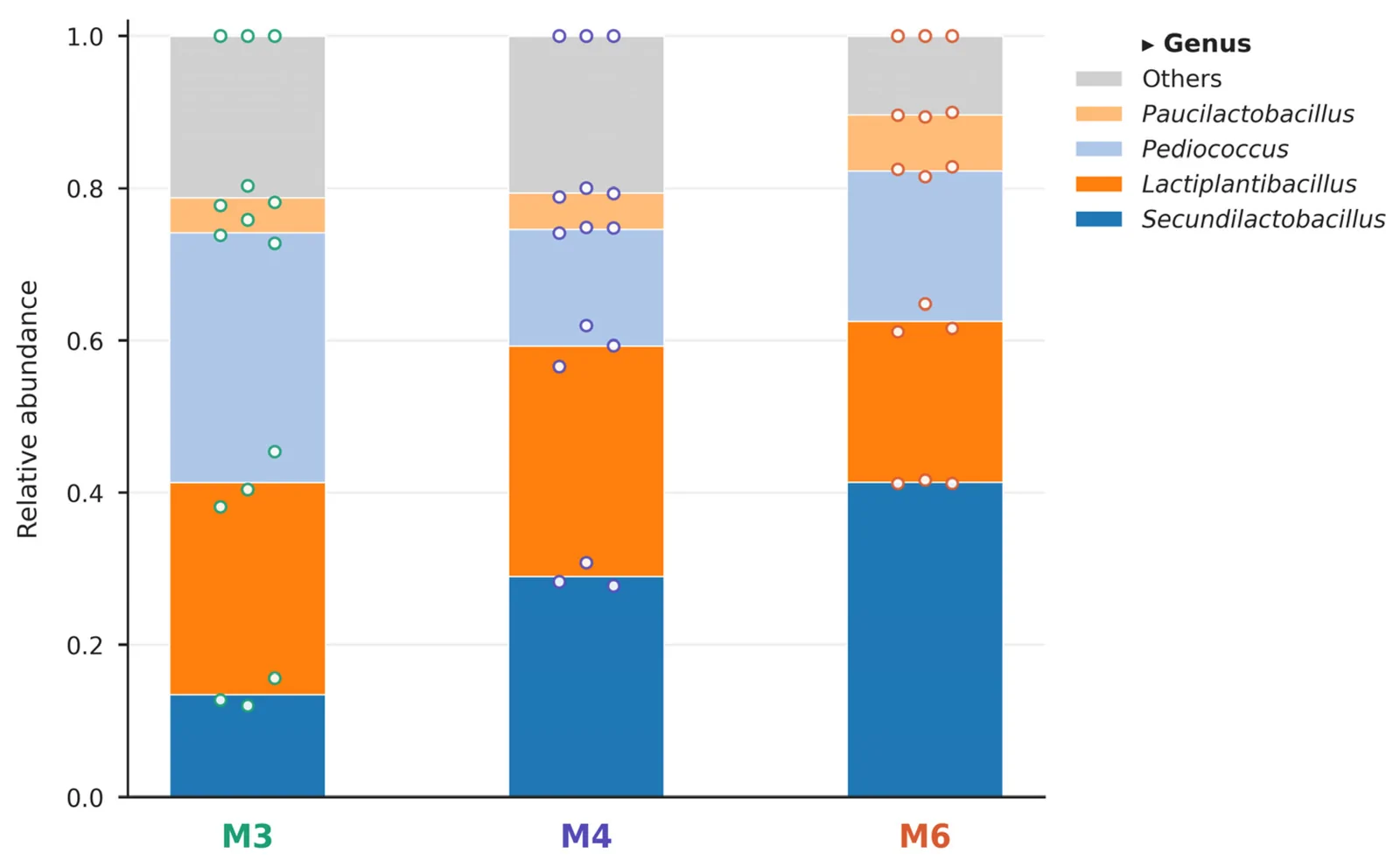
Relative abundance of bacterial genera during the spontaneous fermentation of *Bella di Cerignola* olives determined by long-read 16S metabarcoding. Genera with a relative abundance ≤ 2% are included in the category “Others”. Dots indicate mean values of three biological replicates analysed after 3 (M3), 4 (M4), and 6 (M6) months of fermentation.

After also excluding all taxa outside the family Lactobacillaceae (19%, 17% and 3% of the reads at M3, M4 and M6), bacterial composition still differed among sampling times (R^2^ = 0.94, *p* = 0.028) and the Shannon and Simpson indices still decreased from M3 to M4 and M6 (*p* ≤ 0.001; Tukey’s HSD test, *p* < 0.01), whereas the decrease in richness was smaller (from 28 to 22 species within Lactobacillaceae family) and not consistent among subsamplings. At the genus level, the bacterial community was largely dominated by representatives of the genera *Secundilactobacillus*, *Lactiplantibacillus*, and *Pediococcus*, whereas the remaining taxa constituted only minor fractions (Figure 4).

**Figure 5:**
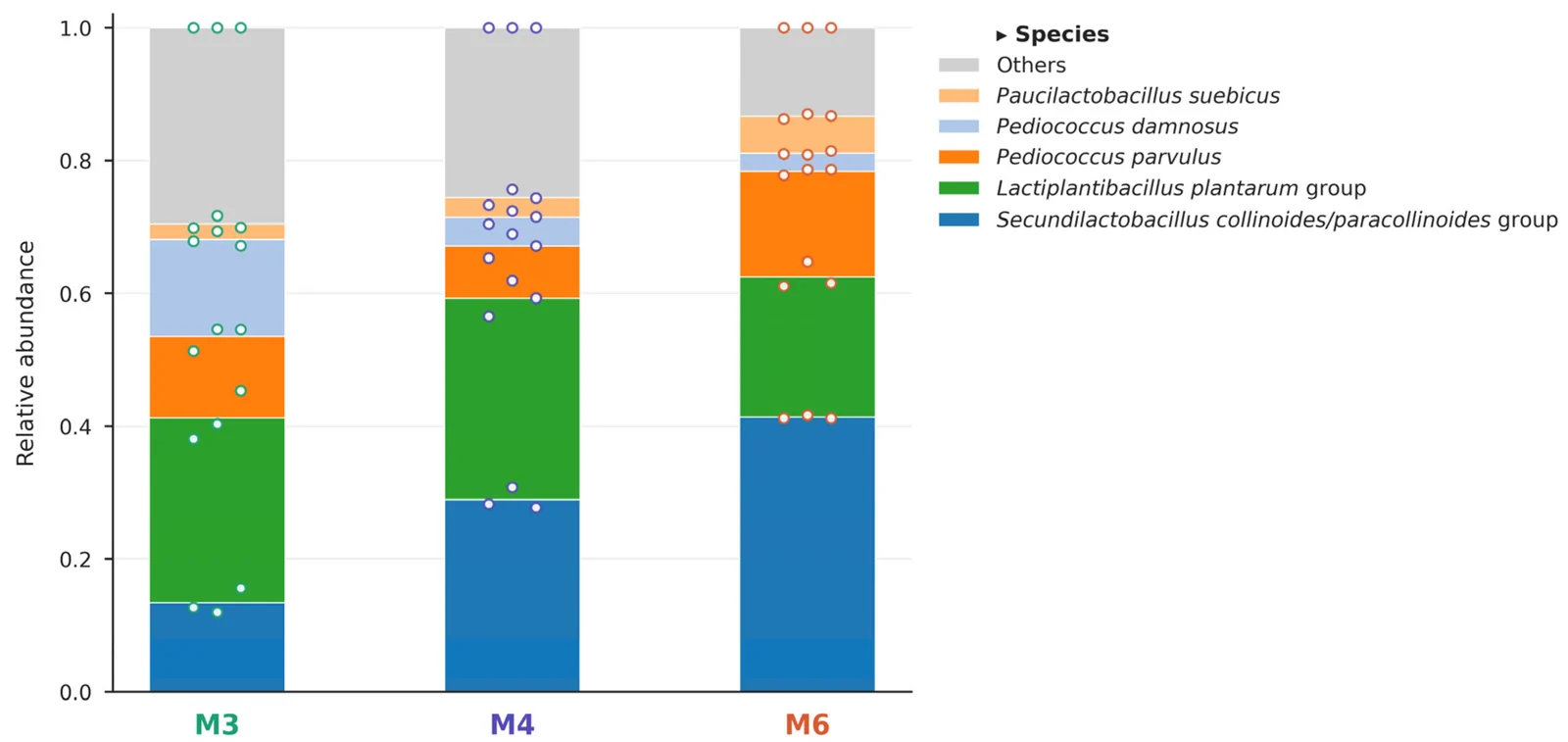
Relative abundance of bacterial species during the spontaneous fermentation of *Bella di Cerignola* olives determined by long-read 16S metabarcoding. Species with a relative abundance ≤ 2% are included in the category “Others”. Dots indicate mean values of three biological replicates analysed after 3 (M3), 4 (M4), and 6 (M6) months of fermentation.

Species-level analysis revealed that the *Secundilactobacillus collinoides*/*paracollinoides* group increased markedly during fermentation, becoming dominant at M4 and M6, whereas the *L. plantarum* group was the most abundant bacterial taxon at M3 (Figure 5). Additional species detected at lower relative abundances included *P. parvulus* and *P. damnosus*. The most notable changes in relative abundance were observed for the *S. collinoides*/*paracollinoides* group, which increased from 13% at M3 to 41% at M6, and *P. damnosus*, which decreased from 15% to 3% over the same period. Moreover, for core microbiome species, agreement of taxonomic assignment between Emu and Kraken2 has been verified (Table S4).

The fungal community associated with the spontaneous fermentation of PDO *Bella di Cerignola* olives was characterised by long-read ITS metabarcoding at 3, 4 and 6 months of fermentation (Table S5).

**Figure 6:**
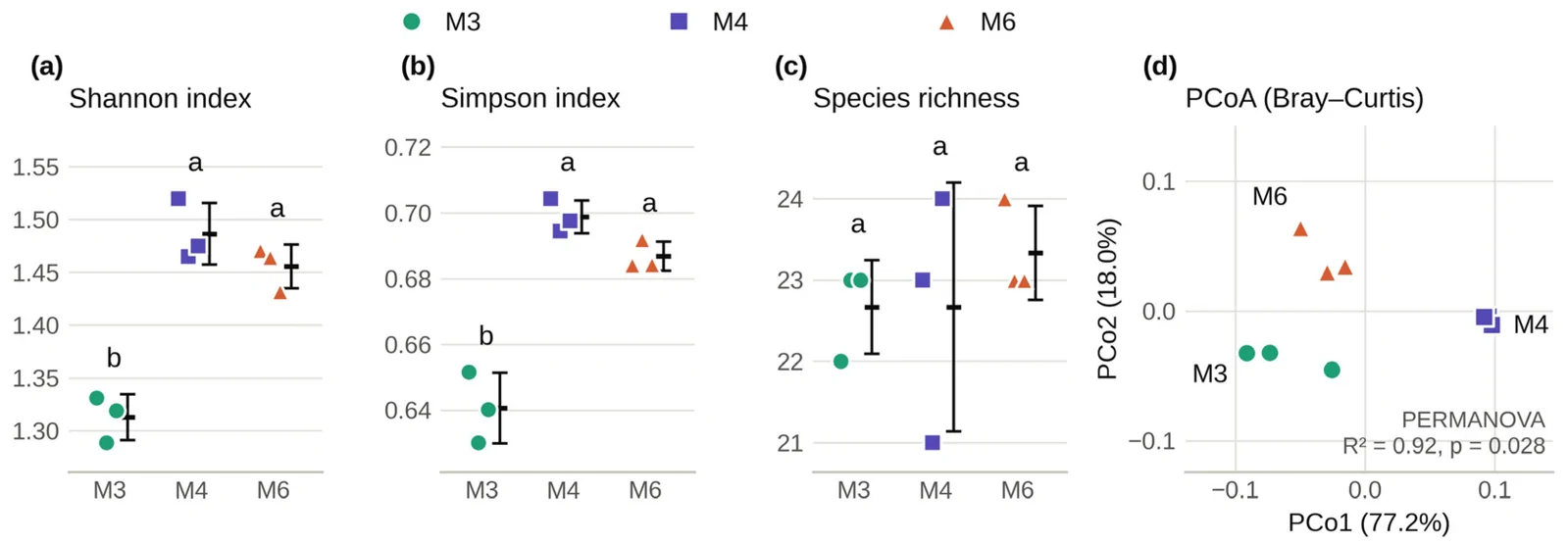
Alpha and beta diversity of the fungal communities during spontaneous fermentation of *Bella di Cerignola* olives at 3 (M3), 4 (M4) and 6 (M6) months. Shannon index, Simpson index and species richness: each symbol is one biological replicate; the horizontal dash and the vertical bar indicate the mean and the standard deviation. Within each panel, time points with different letters are significantly different (repeated-measures ANOVA followed by Tukey’s HSD test, *p* ≤ 0.05) (**a**–**c**). Principal coordinate analysis (PCoA) of Bray–Curtis dissimilarities; each symbol is one sample, and the PERMANOVA result is given in each panel (**d**).

The analysis of fungal alpha diversity (Figure 6a–c) revealed that Shannon and Simpson indices were higher at M4 and M6 than at M3 (repeated-measures ANOVA, *p* < 0.01; Tukey’s HSD test, *p* < 0.01), with no difference between M4 and M6, indicating a more even distribution of abundances after M3, whereas species richness did not change significantly (*p* = 0.69). Analysis of beta diversity (Figure 6d) revealed fungal community composition differed among time points (PERMANOVA, R^2^ = 0.92, *p* = 0.028), although the differences were small (mean Bray–Curtis dissimilarity between time points 0.09–0.16).

**Figure 7:**
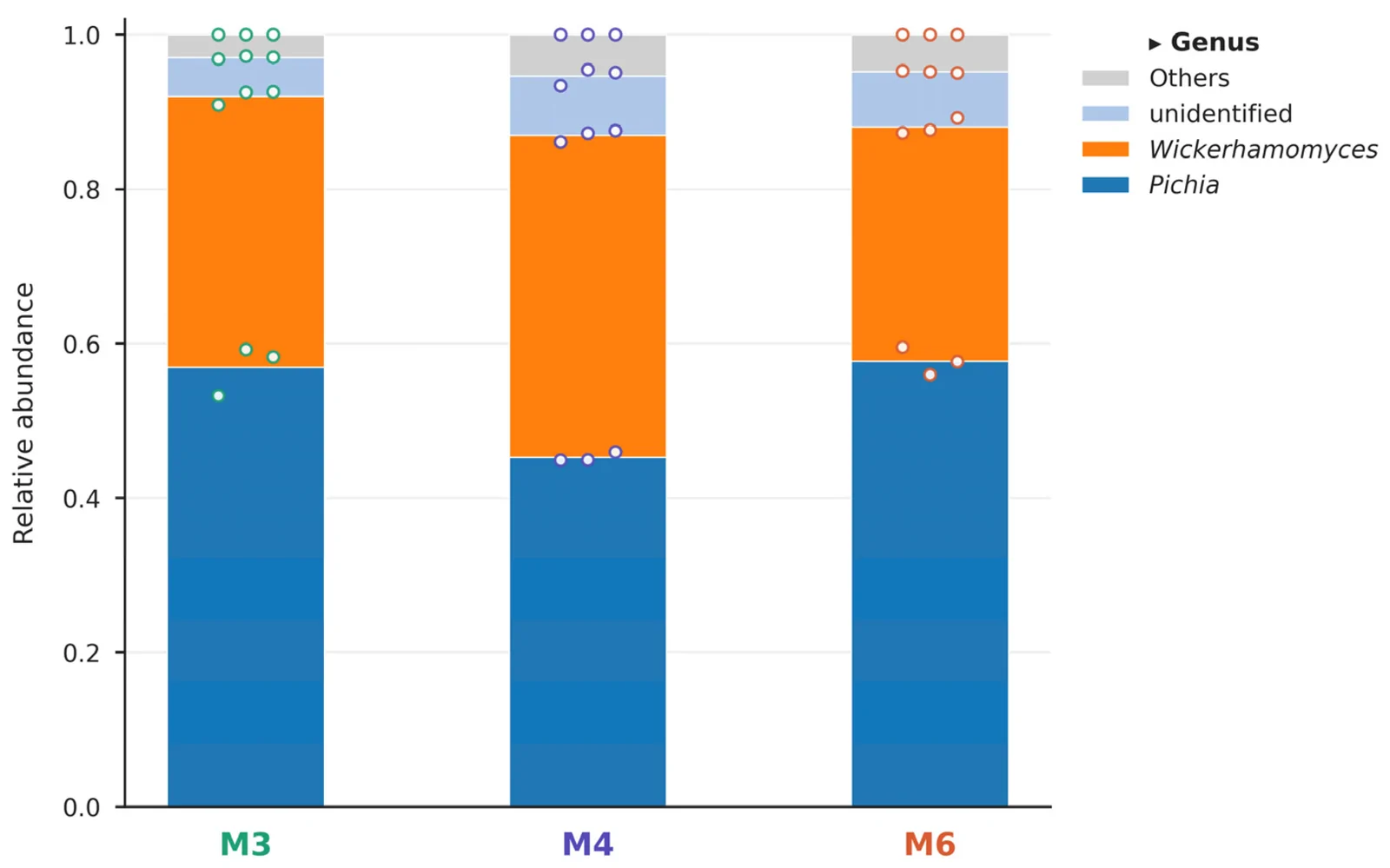
Relative abundance of fungal genera during the spontaneous fermentation of *Bella di Cerignola* olives determined by long-read ITS metabarcoding. Genera with a relative abundance ≤ 2% are included in the category “Others”. Dots indicate mean values of three biological replicates analysed after 3 (M3), 4 (M4), and 6 (M6) months of fermentation.

At the genus level, the fungal community was dominated by *Pichia* and *Wickerhamomyces* during the entire fermentation process, whereas all other taxa occurred at relative abundances below 2% (Figure 7). Only limited changes in taxonomic profiles were observed among sampling times, suggesting the establishment of a stable fungal consortium.

**Figure 8:**
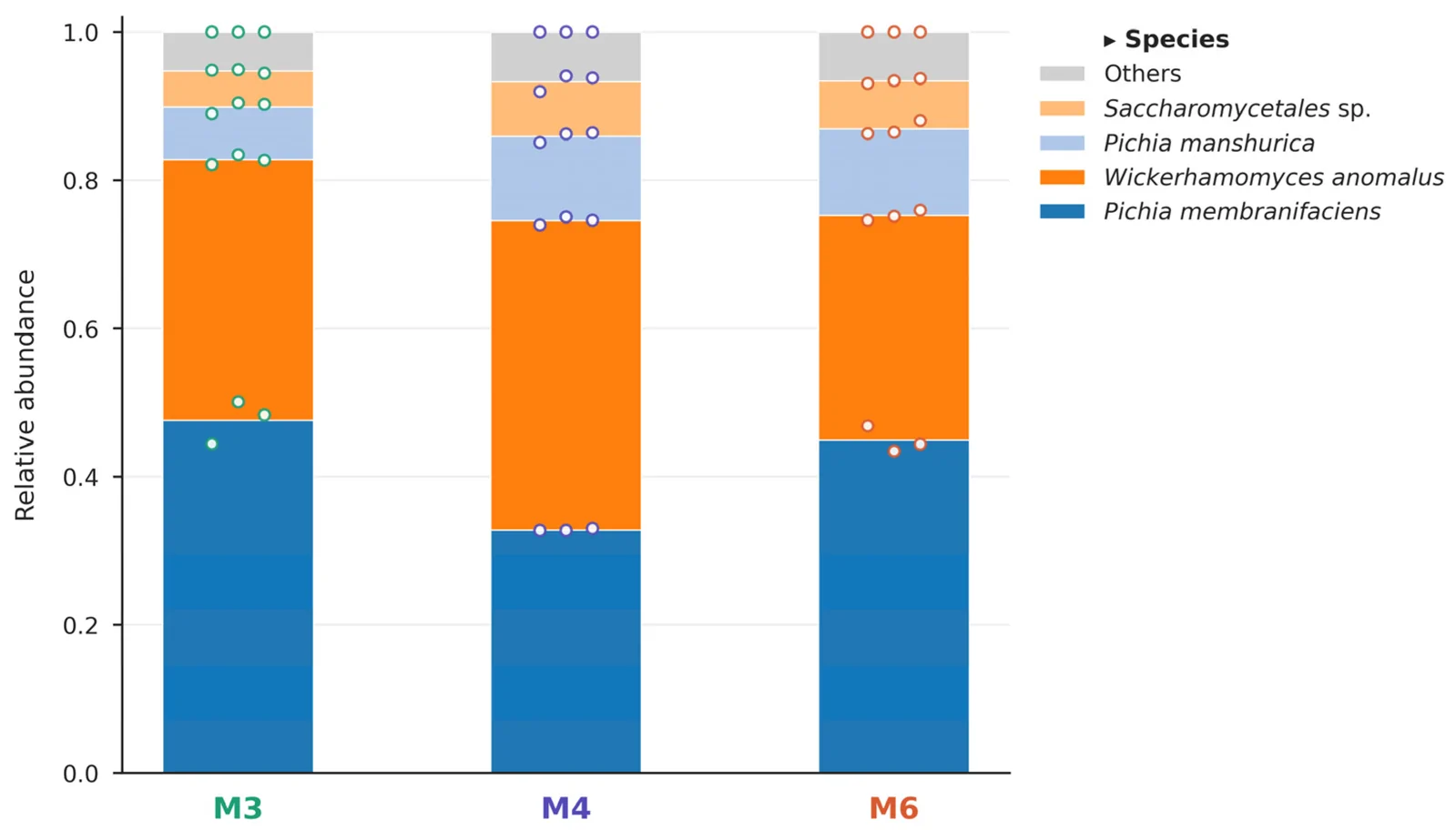
Relative abundance of fungal species during the spontaneous fermentation of *Bella di Cerignola* olives determined by long-read ITS metabarcoding. Species with a relative abundance ≤ 2% are included in the category “Others”. Dots indicate mean values of three biological replicates analysed after 3 (M3), 4 (M4), and 6 (M6) months of fermentation.

Furthermore, species-level analysis confirmed this pattern and revealed that *P. membranifaciens*, *P. manshurica*, and *W. anomalus* represented the dominant fungal species throughout fermentation, although the relative abundance of *P. membranifaciens* slightly decreased at M4 (Figure 8). Minor fractions of reads were assigned to unclassified members of the order *Saccharomycetales* or to other low-abundance taxa, indicating the presence of a residual fungal fraction characterised by limited abundance. Moreover, for core microbiome species, agreement of taxonomic assignment among Emu, Kraken2 and dnabarcoder has been verified (Table S4).

The species-level profiles obtained through long-read metabarcoding were consistent with the results obtained by culture-dependent isolation followed by MALDI-TOF MS identification. Indeed, *P. membranifaciens*, *P. manshurica*, and *W. anomalus* were recovered and identified at all fermentation stages, corroborating the persistence of these taxa throughout the process.

## 4. Discussion

### 4.1. Evolution of Viable Microbial Communities During Spontaneous Fermentation of Bella di Cerignola Olives

The microbial community observed during fermentation reflect the autochthonous microbiota in the absence of starter cultures. The high LAB counts already detected at M3 indicate that the fermentation ecosystem was well established, with LAB representing the dominant microbial group. This pattern is consistent with spontaneous olive fermentations, where LAB and yeasts are commonly reported to interact, contributing to both acidification and development of sensory attributes [2]. In particular, in *Bella di Cerignola* olives, these microbial groups are known to establish a complex and dynamic consortium early during fermentation [5,22]. The stability of LAB populations between M3 and M4 suggests that the fermentation had reached a relatively stable phase. The persistence of high LAB counts is indicative of their successful adaptation to the selective conditions imposed by the brine. Conversely, the decrease in yeast populations observed at M6 may be associated with environmental constraints commonly occurring during olive fermentation, including nutrient depletion, salt stress, metabolite accumulation, and low pH conditions. However, since only pH was directly monitored in the present study, the contribution of these factors should be considered hypothetical and cannot be specifically demonstrated by our data. These conditions represent key ecological drivers in spontaneous fermentations of *Bella di Cerignola*, shaping microbial dynamics and leading to the competitive exclusion of less adapted populations [23].

At M6, the significant differences observed for both yeast populations and bacteria growing on M17 highlight the progressive selective pressure exerted by the fermentation environment. This trend is consistent with the natural progression of spontaneous olive fermentation, where acid-tolerant LAB species, such as *L. plantarum* and *L. pentosus*, predominate over other microbial groups during the final stages, leading to the formation of a simpler and more specialised microbial community [22]. Overall, starting from a relatively diversified microbial consortium, the system evolves toward a more stable ecosystem dominated by LAB, while yeasts undergo a gradual decline. This behaviour is characteristic of natural olive fermentation, where the absence of starter cultures enables the spontaneous microbiota to evolve dynamically under the selective influence of physicochemical parameters such as salinity, pH, and nutrient availability [1,24].

The low pH values recorded throughout the monitored period, together with the persistence of high LAB populations, further suggest that the fermentation process had already reached an advanced stage of ecological stabilisation by the third month. This finding is noteworthy because spontaneous fermentations of *Bella di Cerignola* olives are often reported to acidify slowly and may maintain pH values above 4.2–4.5 during maturation [1,2,10,11]. Therefore, the microbial ecosystem investigated in the present study appears to have reached a mature and stable configuration by the third month of fermentation.

### 4.2. Identification of Culturable Microorganisms by MALDI-TOF MS

MALDI-TOF MS proved to be a valuable tool for the rapid characterisation of the culturable microbiota associated with spontaneous olive fermentation. In recent years, this technique has become increasingly popular in food microbiology due to its rapidity, low operating costs, high-throughput capability, and reliable species-level identification of large collections of isolates [6,25]. Furthermore, MALDI-TOF MS has shown superior discriminatory power compared with conventional identification approaches based solely on 16S rRNA gene sequencing, particularly when closely related species are involved [26].

The predominance of *L. pentosus* observed throughout fermentation is consistent with previous studies on spontaneously fermented table olives. De Angelis et al. [11], studying the microbiota of naturally fermented *Bella di Cerignola* olives, reported *L. pentosus* among the dominant bacterial species during fermentation, highlighting its remarkable adaptation to the olive brine environment. Similar findings were reported by Portilha-Cunha et al. [27], who identified *L. pentosus* and *L. plantarum* as the LAB species most frequently associated with table olive fermentations worldwide. Compared with *L. plantarum*, *L. pentosus* possesses genomic traits associated with carbohydrate utilisation, osmotic stress tolerance, and resistance to phenolic compounds, which may confer a selective advantage under the stressful conditions of olive brines [27,28]. The persistence of *L. pentosus* throughout the six-month fermentation process suggests that this species represents one of the key functional drivers of *Bella di Cerignola* olive fermentation and may constitute a promising candidate for the development of autochthonous starter cultures [11,22].

A particularly relevant finding concerns the differentiation between *L. pentosus* and *L. plantarum*. These species belong to the *L. plantarum* group and share highly similar 16S rRNA gene sequences, often hampering discrimination at the species level. In contrast, MALDI-TOF MS relies on species-specific protein fingerprinting and has been shown to reliably differentiate *L. plantarum*, *L. pentosus*, and *L. paraplantarum* [29]. In the present study, this capability revealed a clear predominance of *L. pentosus*, whereas only a single isolate was assigned to *L. plantarum*, suggesting a higher adaptation of *L. pentosus* to the fermentation conditions of *Bella di Cerignola* olives. Therefore, the bacterial community showed only partial correspondence between the culture-dependent and culture-independent approaches. Such discrepancies underscore the complementary strengths of these methodologies, while also reflecting the biases and limitations associated with each approach. Regarding the yeast population, the community also remained relatively stable throughout fermentation, suggesting the persistence of well-adapted species under the selective conditions imposed by the brine environment, and the recurrent isolation of *P. membranifaciens*, *P. manshurica*, and *W. anomalus* agrees with previous studies on table olive fermentations. Botta and Cocolin [23] reported these genera among the most common yeasts occurring during spontaneous olive fermentation, while De Angelis et al. [11] reported the persistence of *W. anomalus* in fermented *Bella di Cerignola* olives. These species are known to contribute to aroma development, phenolic compound transformation, and positive interactions with LAB, thus playing an important role in the stability and sensory quality of the final product. The simultaneous persistence of *L. pentosus*, *Pediococcus* spp., *W. anomalus* and *Pichia* spp. suggests the establishment of a stable multispecies consortium rather than a simple replacement of one microbial group by another. Such interactions are increasingly recognised as crucial drivers of spontaneous vegetable fermentation. Yeasts provide amino acids, vitamins and other growth-promoting factors that support LAB development, whereas LAB contribute to environmental acidification, thereby limiting the proliferation of undesirable microorganisms [2].

It should be emphasised that the isolates analysed by MALDI-TOF MS were randomly selected from countable plates by choosing well-isolated colonies and, whenever possible, colonies displaying different morphologies. Consequently, the resulting microbial profile should be considered representative of the culturable fraction of the microbiota rather than an exhaustive description of the entire fermentation ecosystem. Differences in cultivability, growth rate, and competitive behaviour on culture media may have favoured the isolation of specific taxa, while low-abundance or slow-growing microorganisms could have remained undetected. Although the recurrent recovery of *L. pentosus*, *P. manshurica*, *P. membranifaciens*, and *W. anomalus* strongly suggests that these microorganisms represent the dominant culturable members of the fermentation microbiota, the presence of additional taxa cannot be excluded. Therefore, the results obtained by MALDI-TOF MS should be interpreted as a description of the dominant culturable microbiota. To overcome the intrinsic limitations of culture-dependent analyses, the present study was complemented by a long-read metabarcoding approach, enabling the investigation of the microbial community directly from environmental DNA. Rather than representing alternative methodologies, MALDI-TOF MS and metabarcoding should be considered complementary approaches. While MALDI-TOF MS provides high-resolution identification of viable and culturable isolates, metabarcoding offers a broader overview of community composition, including taxa that may be underrepresented or undetected using cultivation-based methods. The integration of culture-dependent and culture-independent approaches has been increasingly recognised as an effective strategy for the comprehensive characterisation of microbial ecosystems associated with spontaneous food fermentations, including table olives [1,23,30].

### 4.3. Bacterial Community Revealed by Long-Read 16S Metabarcoding Analysis

The alpha-diversity and β-diversity analyses indicate that the bacterial community, although dominated by the same taxa at all sampling times, changed between M3 and M6. The decrease observed in diversity indices mainly reflected the decline of the taxa outside the Lactobacillaceae, mostly halophilic bacteria, and the increase in the *S. collinoides*/*paracollinoides* group; as no negative extraction controls were included, a contribution of reagent DNA to these minor taxa cannot be excluded. The persistence of the same dominant taxa indicates that the core bacterial community had already been established by the third month of fermentation.

The high abundance of the *S. collinoides*/*paracollinoides* group, the most abundant taxon at M6, is particularly noteworthy. Despite their abundance in the metabarcoding data, these species were not recovered among the isolates identified by MALDI-TOF MS. This discrepancy likely reflects the inherent differences between culture-dependent and culture-independent approaches. While metabarcoding characterises microbial communities directly from total DNA, MALDI-TOF MS analysis relies on the successful cultivation and isolation of individual colonies.

Previous studies have reported that *S. paracollinoides* can be particularly difficult to recover using conventional culture media, as its growth performance is strongly influenced by physiological adaptation, previous subculture history, and culture conditions, including medium composition and pH. In particular, Shimokawa and Suzuki [31] demonstrated that certain *S. paracollinoides* strains failed to grow on conventional media such as MRS agar or exhibited extremely slow growth, despite remaining viable, and that cultivability could vary markedly depending on prior acclimatisation conditions. Such characteristics may explain why this species was not isolated under the cultivation conditions adopted in the present study despite its high abundance in the sequencing dataset. Moreover, the relatively low representation of *S. paracollinoides* and *S. collinoides* strains in international microbial culture collections suggests that these species may be systematically overlooked during conventional isolation procedures, likely due to their demanding growth requirements and variable cultivability (World Data Centre for Microorganisms, https://gcm.wdcm.org, accessed on 4 September 2026).

The predominance of the *S. collinoides*/*paracollinoides* group observed here is noteworthy because these species have been reported in olive fermentations mainly through culture-independent analyses rather than conventional isolation-based studies. Its occurrence in naturally fermented *Nocellara Etnea* olives [32] and in the microbiome of *Kalamata* olives [33] was revealed by sequencing approaches, similarly to the strategy adopted in the present work. These findings support the hypothesis that *S. collinoides*/*paracollinoides* strains are important members of the bacterial community associated with olive fermentation and highlight the value of culture-independent approaches in revealing taxa that may remain undetected through conventional isolation procedures.

Long-read metabarcoding also revealed a substantial abundance of the *L. plantarum* group throughout the fermentation process. It is important to emphasise that long-read metabarcoding is based on the amplification and sequencing of the 16S rRNA gene, a widely used phylogenetic marker for bacterial community analysis. However, members of the *L. plantarum* group, including *L. plantarum*, *L. pentosus* and *L. paraplantarum*, shared highly similar 16S rRNA gene sequences, which may limit species-level discrimination and potentially lead to ambiguous taxonomic assignments. Although full-length 16S sequencing improves taxonomic resolution compared with short-read approaches, discrimination among members of the *L. plantarum* group remains challenging. Therefore, confirmation at species level would require additional genetic markers such as recA, pheS, or whole-genome-based approaches [34]. In contrast, MALDI-TOF MS identifies microorganisms through species-specific protein fingerprint profiles and has proven effective for the identification of lactobacilli at the species level [35], including the discrimination of closely related members of the *L. plantarum* group such as *L. plantarum* and *L. pentosus* [29]. Indeed, the observed discrepancy highlights the added value of integrating culture-independent and culture-dependent approaches. If metabarcoding data had been considered alone, the dominant taxon would have been interpreted as the *L. plantarum* group. The additional MALDI-TOF MS characterisation of isolates provided further taxonomic resolution within the *L. plantarum* group and suggested that *L. pentosus* may represent the predominant species among the culturable fraction. Besides the *S. collinoides*/*paracollinoides* and *L. plantarum* groups, the long-read metabarcoding analysis also revealed the presence of *P. parvulus* and *P. damnosus* throughout fermentation, further highlighting the complexity of the bacterial ecosystem associated with *Bella di Cerignola* fermentation. Both species have previously been reported in spontaneous table olive fermentations and are recognised as members of the LAB consortium adapted to brine environments. In particular, *P. parvulus* has been reported in naturally fermented table olives of different cultivars, including *Tonda di Cagliari*, and has been identified among the dominant LAB populations by culture-independent approaches [36,37]. Likewise, species belonging to the genus *Pediococcus*, including *P. damnosus*, have repeatedly been identified in spontaneous olive fermentations and are recognised as contributors to the establishment and stabilisation of the fermentative microbiota [11,27].

Overall, the long-read metabarcoding analysis revealed a bacterial community dominated by the *S. collinoides*/*paracollinoides* and *L. plantarum* groups, while also identifying less abundant LAB taxa that contribute to community structure and stability. The comparison with MALDI-TOF MS further emphasises the complementary nature of culture-independent and culture-dependent approaches, highlighting the importance of integrating both methodologies to obtain a comprehensive characterisation of microbial diversity during spontaneous olive fermentation.

### 4.4. Fungal Community Revealed by Long-Read ITS Metabarcoding Analysis

The fungal community of spontaneously fermented PDO *Bella di Cerignola* olives exhibited remarkable temporal stability throughout the fermentation process. Both alpha- and beta-diversity analyses indicated only minor changes between sampling times, suggesting that the fungal ecosystem rapidly reached a stable configuration capable of persisting under the selective conditions imposed by the olive brine environment.

The predominance of *P. membranifaciens*, *P. manshurica*, and *W. anomalus* is consistent with previous investigations on *Bella di Cerignola* table olives, where yeasts belonging to the genera *Pichia* and *Wickerhamomyces* were reported among the dominant fungal populations during spontaneous fermentation [11,38]. The recurrent detection of these species across all sampling times suggests the establishment of a core yeast consortium well adapted to the environmental conditions of olive brine and nutrient availability.

Among the dominant taxa detected in this study, *P. membranifaciens* has previously been reported during the spontaneous fermentation of *Cellina di Nardò* and *Leccino* olives, demonstrating its ability to persist under conditions characterised by elevated salt concentrations and progressive acidification [39]. Similarly, *P. manshurica* has been identified as a relevant component of the microbiota associated with naturally fermented Italian table olives, indicating its widespread occurrence in olive fermentation ecosystems [5]. The ecological relevance of *W. anomalus* has also been extensively documented. This species was identified as a dominant member of the fungal community in fermented table olives from Cyprus through metabarcoding approaches [40] and has also been reported in naturally fermented Italian olives [39]. The repeated occurrence of *W. anomalus* in geographically and technologically distinct olive fermentations suggests remarkable ecological adaptability and a key role in establishing the fermentation microbiota.

Beyond their persistence in olive brines, these species are considered particularly adapted to stressful fermentation environments characterised by high salinity, low pH, and reduced nutrient availability. Moreover, they have been associated with desirable technological properties, including the production of volatile aroma compounds, the transformation of phenolic substrates, and positive interactions with LAB, all of which may contribute to the microbial stability of the system and to the development of the sensory characteristics of the final product [11,38]. A notable finding of the present study was the overall concordance between culture-dependent and culture-independent approaches for fungal community characterisation. The dominant yeast species identified by long-read ITS metabarcoding were consistently recovered through cultivation and subsequently identified by MALDI-TOF MS. Although metabarcoding provided a broader overview of the microbial community composition, including additional abundant taxa not readily recovered by cultivation, the agreement observed for the predominant yeasts supports the robustness and complementarity of the two approaches. These results indicate that *P. membranifaciens*, *P. manshurica*, and *W. anomalus* represent key members of the fungal microbiota of naturally fermented *Bella di Cerignola* olives, showing persistence throughout the fermentation process.

## 5. Conclusions

The spontaneous fermentation of PDO *Bella di Cerignola* olives was characterised by the establishment of a stable and highly adapted microbial ecosystem dominated by LAB and supported by a persistent core yeast community. Throughout the investigated period of the fermentation process, LAB remained the predominant microbial group, whereas yeasts showed a progressive decline during the final stages, reflecting the increasing selective pressure imposed by the fermentation environment. Long-read metabarcoding revealed that the bacterial community, although dominated by the same taxa at all sampling times, changed between M3 and M6. The *S. collinoides*/*paracollinoides* and *L. plantarum* groups were identified as the dominant bacterial taxa, while *P. membranifaciens*, *P. manshurica*, and *W. anomalus* consistently represented the core fungal microbiota. The recurrent detection of these microorganisms throughout fermentation suggests that they are key ecological players in the spontaneous fermentation of *Bella di Cerignola* olives and may contribute substantially to fermentation stability and final product quality.

The integrated use of MALDI-TOF MS and long-read metabarcoding provided complementary insights into the microbial ecology of *Bella di Cerignola* olives. While MALDI-TOF MS identified *L. pentosus* as the predominant culturable LAB species, metabarcoding revealed the abundance of the *S. collinoides*/*paracollinoides* group, a taxon that was not recovered under the cultivation conditions employed. This discrepancy highlights the limitations of culture-dependent approaches and demonstrates the added value of sequencing-based methods for obtaining a more comprehensive view of olive fermentation microbiota. Particularly noteworthy was the consistent detection of the *S. collinoides*/*paracollinoides* group throughout fermentation. Since this species has been reported in olive fermentations mainly through culture-independent studies, its prevalence in the present work suggests that it may represent an overlooked but ecologically relevant member of the olive microbiome whose physiological role and technological potential deserve further investigation.

Overall, this study expands current knowledge of the microbial ecology associated with PDO *Bella di Cerignola* olives and demonstrates that combining culture-dependent and culture-independent approaches is an effective strategy for identifying both culturable and previously overlooked microbial populations. Future studies should focus on the phenotypic, technological, and safety characterisation of selected autochthonous isolates and on their evaluation in controlled fermentation trials to assess their suitability as starter cultures for *Bella di Cerignola* table olive production. Further investigations should include fresh fruits and early fermentation stages to provide a more complete characterisation of microbial community establishment and succession. Moreover, integration of metagenomics, metabolomics and volatile analysis will be necessary to elucidate the functional role of dominant microbial taxa during *Bella di Cerignola* fermentation.

## Data Availability

The sequence data are available at NCBI SRA under BioProject ID: PRJNA1525609.

## Supplementary Materials

The following supporting information can be downloaded at https://www.mdpi.com/article/10.3390/foods15203685/s1, Table S1: Number of bacterial and yeast isolates picked per each medium (MRS, M17 and DRBC) at 3 (M3), 4 (M4), and 6 (M6) months of spontaneous fermentation of *Bella di Cerignola* olives and identified by MALDI-TOF MS. Table S2: Total reads yield and mean read length distribution per sample. Table S3: ASV table of bacterial population. Table S4: Comparison of taxonomical assignment for core microbiome species belonging to Emu, Kraken2 and dnabarcoder taxonomic classifiers. Taxonomic identification at genus e/o species level is indicated as assigned (+) or not available (n.a.). Table S5: ASV table of fungal population.
