# Supplementary material for: Unveiling the Microbial Community of Naturally Fermented Bella di Cerignola PDO Table Olives Integrating Culture-Dependent, MALDI-TOF MS Identification and Long-Read Metabarcoding Approaches
Source: Foods. 2026 Oct 16;15(20):3685. doi: 10.3390/foods15203685 (PMC13704255; doi:10.3390/foods15203685)
Supplement: Supplementary file 1 [file foods-15-03685-s001.zip › foods-4590525-supplementary.pdf]

Table S1. Number of **b**acterial and yeast isolates picked per each medium (MRS, M17 and DRBC) at 3 (M3), 4 (M4), and 6 (M6) months of spontaneous fermentation of *Bella di Cerignola* olives identified by MALDI-TOF MS.

Colony ID	Sampling time	Growth medium	Identified species	MALDI-TOF MS score [a]
1	M3	MRS	*Lactiplantibacillus pentosus*	2.43
2	M3	MRS	*Lactiplantibacillus pentosus*	2.44
3	M3	MRS	*Lactiplantibacillus pentosus*	2.45
4	M3	MRS	*Lactiplantibacillus pentosus*	2.41
5	M3	MRS	*Lactiplantibacillus pentosus*	2.45
6	M3	MRS	*Lactiplantibacillus pentosus*	2.39
7	M3	MRS	*Lactiplantibacillus pentosus*	2.39
8	M3	MRS	*Lactiplantibacillus pentosus*	2.45
9	M3	MRS	*Lactiplantibacillus pentosus*	2.46
10	M3	MRS	*Lactiplantibacillus pentosus*	2.38
11	M3	MRS	*Lactiplantibacillus pentosus*	2.42
12	M3	MRS	*Lactiplantibacillus pentosus*	2.40
13	M3	MRS	*Lactiplantibacillus pentosus*	2.41
14	M3	M17	*Pediococcus parvulus*	2.22
15	M3	M17	*Pediococcus parvulus*	2.13
16	M3	M17	*Pediococcus ethanolidurans*	2.01 *
17	M3	M17	*Pediococcus parvulus*	2.09
18	M3	M17	*Pediococcus ethanolidurans*	2.05
19	M3	M17	*Pediococcus parvulus*	2.09
20	M3	M17	*Pediococcus parvulus*	2.04 *
21	M3	DRBC	*Pichia membranifaciens*	2.05
22	M3	DRBC	*Pichia manshurica*	2.36
23	M3	DRBC	*Pichia manshurica*	2.34
24	M3	DRBC	*Wickerhamomyces anomalus*	2.15
25	M3	DRBC	*Pichia membranifaciens*	2.09
26	M3	DRBC	*Wickerhamomyces anomalus*	2.18
27	M3	DRBC	*Pichia membranifaciens*	2.06
28	M3	DRBC	*Wickerhamomyces anomalus*	2.20
29	M3	DRBC	*Wickerhamomyces anomalus*	2.13
30	M3	DRBC	*Pichia manshurica*	2.35
31	M3	DRBC	*Pichia membranifaciens*	2.07
32	M3	DRBC	*Pichia membranifaciens*	2.17
33	M3	DRBC	*Pichia membranifaciens*	2.18
34	M3	DRBC	*Pichia membranifaciens*	2.22
35	M4	MRS	*Lactiplantibacillus pentosus*	2.05
36	M4	MRS	*Lactiplantibacillus pentosus*	2.12
37	M4	MRS	*Lactiplantibacillus pentosus*	2.09
38	M4	MRS	*Lactiplantibacillus pentosus*	2.27
39	M4	MRS	*Lactiplantibacillus pentosus*	2.23
40	M4	MRS	*Lactiplantibacillus pentosus*	2.38
41	M4	MRS	*Lactiplantibacillus pentosus*	2.19
42	M4	MRS	*Lactiplantibacillus pentosus*	2.15
43	M4	MRS	*Lactiplantibacillus pentosus*	2.20

44	M4	MRS	*Lactiplantibacillus pentosus*	2.13
45	M4	MRS	*Lactiplantibacillus plantarum*	2.19
46	M4	M17	*Pediococcus ethanolidurans*	2.02 *
47	M4	M17	*Pediococcus parvulus*	2.04 *
48	M4	DRBC	*Pichia manshurica*	2.31
49	M4	DRBC	*Wickerhamomyces anomalus*	2.04 *
50	M4	DRBC	*Wickerhamomyces anomalus*	2.11
51	M4	DRBC	*Pichia manshurica*	2.18
52	M4	DRBC	*Wickerhamomyces anomalus*	2.02 *
53	M4	DRBC	*Wickerhamomyces anomalus*	2.07
54	M4	DRBC	*Wickerhamomyces anomalus*	2.17
55	M4	DRBC	*Wickerhamomyces anomalus*	2.06
56	M4	DRBC	*Pichia membranifaciens*	2.09
57	M4	DRBC	*Pichia membranifaciens*	2.05
58	M4	DRBC	*Pichia manshurica*	2.41
59	M6	M17	*Lactiplantibacillus pentosus*	2.13
60	M6	M17	*Pediococcus parvulus*	2.23
61	M6	M17	*Pediococcus parvulus*	2.34
62	M6	M17	*Pediococcus parvulus*	2.22
63	M6	M17	*Pediococcus parvulus*	2.29
64	M6	MRS	*Lactiplantibacillus pentosus*	2.12
65	M6	MRS	*Lactiplantibacillus pentosus*	2.05
66	M6	MRS	*Lactiplantibacillus pentosus*	2.12
67	M6	MRS	*Lactiplantibacillus pentosus*	2.21
68	M6	MRS	*Lactiplantibacillus pentosus*	2.09
69	M6	MRS	*Lactiplantibacillus pentosus*	2.28
70	M6	MRS	*Lactiplantibacillus pentosus*	2.24
71	M6	MRS	*Lactiplantibacillus pentosus*	2.08
72	M6	MRS	*Lactiplantibacillus pentosus*	2.15
73	M6	MRS	*Lactiplantibacillus pentosus*	2.38
74	M6	MRS	*Lactiplantibacillus pentosus*	2.11
75	M6	MRS	*Lactiplantibacillus pentosus*	2.10
76	M6	DRBC	*Wickerhamomyces anomalus*	2.20
77	M6	DRBC	*Pichia membranifaciens*	2.06
78	M6	DRBC	*Pichia membranifaciens*	2.06
79	M6	DRBC	*Pichia membranifaciens*	2.03 *
80	M6	DRBC	*Pichia manshurica*	2.31
81	M6	DRBC	*Pichia membranifaciens*	2.02 *
82	M6	DRBC	*Pichia manshurica*	2.32
83	M6	DRBC	*Pichia membranifaciens*	2.05
84	M6	DRBC	*Pichia membranifaciens*	2.21
85	M6	DRBC	*Wickerhamomyces anomalus*	2.01 *
86	M6	DRBC	*Wickerhamomyces anomalus*	2.02 *
87	M6	DRBC	*Pichia manshurica*	2.38

[a] score ≥ 2.00 indicates high confidence identification at the species level, according to the Bruker MALDI Biotyper manufacturer's criteria. Borderline identification scores (< 2.05) are flagged with an asterisk (*).

Table S2. Total reads yield and mean read length distribution per sample.

Sample (SRA library ID)	Marker	Sampling time	Reads (Q>10)	Mean read length (bp)
M3ITSa	ITS region	M3	411,558	471
M3ITSb	ITS region	M3	582,434	467
M3ITSc	ITS region	M3	676,255	468
M4ITSa	ITS region	M4	185,034	602
M4ITSb	ITS region	M4	101,461	602
M4ITSc	ITS region	M4	207,157	601
M6ITSa	ITS region	M6	103,171	467
M6ITSb	ITS region	M6	63,766	472
M6ITSc	ITS region	M6	185,970	468
M316Sa	16S rRNA gene	M3	328,731	1,513
M316Sb	16S rRNA gene	M3	940,725	1,512
M316Sc	16S rRNA gene	M3	595,782	1,512
M416Sa	16S rRNA gene	M4	610,200	1,512
M416Sb	16S rRNA gene	M4	696,834	1,512
M416Sc	16S rRNA gene	M4	602,602	1,512
M616Sa	16S rRNA gene	M6	595,795	1,513
M616Sb	16S rRNA gene	M6	428,649	1,513
M616Sc	16S rRNA gene	M6	647,901	1,513

Table S3. ASV table of bacterial population.

	M316Sa	M316Sb	M316Sc	M416Sa	M416Sb	M416Sc	M616Sa	M616Sb	M616Sc	Kingdom	Phylum	Class	Order	Family	Genus	Species
seq1	41648	112341	92773	172208	214237	166961	245264	178385	266727	Bacteria	Bacillota	Bacilli	Lactobacillales	Lactobacillaceae	Lactobacillus	paracollinoides
seq2	77986	247018	165022	167471	209121	183569	114240	96114	128032	Bacteria	Bacillota	Bacilli	Lactobacillales	Lactobacillaceae	Lactiplantibacillus	plantarum
seq3	43419	133801	54834	53536	48852	47241	99263	59402	110685	Bacteria	Bacillota	Bacilli	Lactobacillales	Lactobacillaceae	Pediococcus	parvulus
seq4	54248	138978	75158	31080	24019	26473	19308	9646	18227	Bacteria	Bacillota	Bacilli	Lactobacillales	Lactobacillaceae	Pediococcus	damnosus
seq5	21047	55786	35851	37616	36930	32099	7452	4810	8534	Bacteria	Pseudomonadota	Gammaproteobacteria	Alteromonadales	Shewanellaceae	Shewanella	mangrovi
seq6	6603	21771	16297	17486	22841	17018	31303	26245	33900	Bacteria	Bacillota	Bacilli	Lactobacillales	Lactobacillaceae	Lactobacillus	suebicus
seq7	2068	4933	4761	11328	14134	10284	28957	23820	32381	Bacteria	Bacillota	Bacilli	Lactobacillales	Lactobacillaceae	Lactobacillus	parafarraginis
seq8	13587	34076	22066	9370	6644	7765	6673	1751	5869	Bacteria	Bacillota	Bacilli	Lactobacillales	Lactobacillaceae	Pediococcus	cellicola
seq9	6115	19842	15539	11150	12722	10091	11028	7320	12252	Bacteria	Bacillota	Bacilli	Lactobacillales	Lactobacillaceae	Lactobacillus	vaccinostercus
seq10	11250	18385	10719	11894	6588	7121	2239	728	2392	Bacteria	Pseudomonadota	Gammaproteobacteria	Cardiobacteriales	Cardiobacteriaceae	Suttonella	ornithocola
seq11	5383	19713	11971	4911	7602	6238	4191	2862	3668	Bacteria	Bacillota	Bacilli	Lactobacillales	Lactobacillaceae	Lactobacillus	pentosus
seq12	1184	1618	7057	11997	9942	10954	1502	701	2452	Bacteria	Bacillota	Bacilli	Lactobacillales	Lactobacillaceae	Pediococcus	inopinatus
seq13	1406	3816	3276	4522	5674	4465	8177	6623	8366	Bacteria	Bacillota	Bacilli	Lactobacillales	Lactobacillaceae	Lactobacillus	buchneri
seq14	5386	12619	9066	5414	6067	4445	1084	746	1148	Bacteria	Pseudomonadota	Gammaproteobacteria	Oceanospirillales	Halomonadaceae	Salinicola	salarius
seq15	1824	1959	4656	6354	11369	11734	3348	1942	1570	Bacteria	Cyanobacteriota		Nostocales	Symphyonemataceae	Loriellopsis	cavernicola
seq16	3502	10159	7194	5930	6058	4645	799	512	1057	Bacteria	Pseudomonadota	Gammaproteobacteria	Vibrionales	Vibrionaceae	Vibrio	tritonius
seq17	915	820	3274	6302	10897	14534	1251	726	635	Bacteria	Cyanobacteriota		Oscillatoriales	Gomontiellaceae	Crinalium	epipsammum
seq18	4731	24401	3782	882	624	706	250	111	261	Bacteria	Bacillota	Bacilli	Lactobacillales	Lactobacillaceae	Pediococcus	ethanolidurans
seq19	3172	9839	8024	3293	4501	3536	220	189	235	Bacteria	Bacillota	Bacilli	Lactobacillales	Carnobacteriaceae	Marinilactibacillus	sp. 15R
seq20	1683	6117	4799	3571	4296	3132	2558	2033	2840	Bacteria	Bacillota	Bacilli	Lactobacillales	Lactobacillaceae	Lactobacillus	rapi
seq21	2211	3997	4264	2899	3725	2644	217	173	241	Bacteria	Pseudomonadota	Gammaproteobacteria	Vibrionales	Vibrionaceae	Vibrio	sp. HBUAS61001
seq22	1638	3315	3039	2897	3140	2380	691	493	794	Bacteria	Pseudomonadota	Gammaproteobacteria	Oceanospirillales	Oceanospirillaceae	Marinobacterium	mangrovicola
seq23	1470	5081	3064	2706	2707	2064	384	263	409	Bacteria	Pseudomonadota	Gammaproteobacteria	Oceanospirillales		Terasakiispira	papahanaumokuakeensis
seq24	1725	3626	3261	2300	2253	1853	177	106	205	Bacteria	Pseudomonadota	Gammaproteobacteria	Oceanospirillales	Halomonadaceae	Salinicola	zeshunii
seq25	1716	4410	1579	2573	1535	1716	272	102	411	Bacteria	Pseudomonadota	Gammaproteobacteria	Cardiobacteriales	Cardiobacteriaceae	Suttonella	indologenes
seq26	551	1512	1338	2524	3142	1923	573	471	678	Bacteria	Bacillota	Bacilli	Lactobacillales	Lactobacillaceae	Lactobacillus	coryniformis
seq27	924	2500	1621	1415	1709	1361	169	210	211	Bacteria	Bacillota	Bacilli	Lactobacillales	Leuconostocaceae	Leuconostoc	mesenteroides
seq28	632	3371	1065	728	238	501	468	70	428	Bacteria	Bacillota	Bacilli	Lactobacillales	Lactobacillaceae	Lactobacillus	sp. CBA3606
seq29	1015	3289	1133	358	420	276	130	91	118	Bacteria	Pseudomonadota	Gammaproteobacteria			Neiella	marina
seq30	550	1377	997	968	926	825	164	94	166	Bacteria	Pseudomonadota	Gammaproteobacteria		Celerinatantimonadaceae	Celerinatantimonas	diazotrophica
seq31	436	1109	705	1011	983	841	175	115	220	Bacteria	Pseudomonadota	Gammaproteobacteria	Alteromonadales	Idiomarinaceae	Idiomarina	loihiensis
seq32	560	1858	1002	584	665	500	50	53	75	Bacteria	Pseudomonadota	Gammaproteobacteria	Alteromonadales	Idiomarinaceae	Idiomarina	tyrosinivorans
seq33	220	583	605	840	878	1383	259	85	83	Bacteria	Cyanobacteriota		Chroococcales	Microcystaceae	Microcystis	aeruginosa
seq34	497	1837	1159	567	283	352	31	0	46	Bacteria	Pseudomonadota	Gammaproteobacteria	Vibrionales	Vibrionaceae	Vibrio	tubiashii
seq35	521	1471	1139	682	412	454	21	14	25	Bacteria	Pseudomonadota	Gammaproteobacteria	Oceanospirillales	Halomonadaceae	Salinicola	tamaricis
seq36	491	1809	973	382	175	272	30	0	23	Bacteria	Pseudomonadota	Gammaproteobacteria	Alteromonadales	Shewanellaceae	Parashewanella	tropica
seq37	204	693	494	565	808	527	91	99	132	Bacteria	Bacillota	Bacilli	Lactobacillales	Leuconostocaceae	Weissella	paramesenteroides
seq38	167	308	506	613	610	1023	178	71	71	Bacteria	Cyanobacteriota		Oscillatoriales	Oscillatoriaceae	Oscillatoria	nigro-viridis
seq39	235	510	404	454	464	377	88	45	89	Bacteria	Pseudomonadota	Gammaproteobacteria	Alteromonadales	Alteromonadaceae	Marinobacter	oulmenensis
seq40	226	953	499	288	288	218	62	42	38	Bacteria	Pseudomonadota	Gammaproteobacteria	Oceanospirillales	Oceanospirillaceae	Marinobacterium	zhoushanense
seq41	295	705	589	380	255	269	30	18	20	Bacteria	Pseudomonadota	Gammaproteobacteria	Vibrionales	Vibrionaceae	Salinivibrio	kushneri
seq42	158	1082	361	180	123	148	29	0	23	Bacteria	Pseudomonadota	Gammaproteobacteria	Vibrionales	Vibrionaceae	Vibrio	furnissii
seq43	123	223	316	366	489	324	67	30	75	Bacteria	Bacteroidota	Flavobacteriia	Flavobacteriales	Flavobacteriaceae	Zunongwangia	profunda
seq44	175	588	327	231	115	187	23	18	12	Bacteria	Pseudomonadota	Gammaproteobacteria	Oceanospirillales	Oceanospirillaceae	Neptunomonas	acidivorans
seq45	205	597	355	177	114	106	21	0	0	Bacteria	Pseudomonadota	Gammaproteobacteria	Oceanospirillales	Halomonadaceae	Salinicola	halophilus
seq46	96	174	195	143	191	159	218	168	222	Bacteria	Bacillota	Bacilli	Lactobacillales	Lactobacillaceae	Lactobacillus	kisonensis
seq47	57	193	127	193	224	178	192	158	167	Bacteria	Bacillota	Bacilli	Lactobacillales	Lactobacillaceae	Lactobacillus	collinoides
seq48	64	93	142	246	311	411	72	60	38	Bacteria	Cyanobacteriota		Chroococcales	Chroococcaceae	Gloeocapsa	sp. PCC 7428
seq49	122	336	229	205	127	132	15	0	18	Bacteria	Pseudomonadota	Gammaproteobacteria	Oceanospirillales	Oceanospirillaceae	Marinobacterium	litorale
seq50	51	295	111	95	92	91	166	81	193	Bacteria	Bacillota	Bacilli	Lactobacillales	Lactobacillaceae	Lactobacillus	plajomi
seq51	67	74	133	221	246	337	41	29	21	Bacteria	Cyanobacteriota		Chroococcidiopsidales	Chroococcidiopsidaceae	Chroococcidiopsis	thermalis
seq52	77	126	149	189	170	310	48	26	24	Bacteria	Cyanobacteriota		Nostocales	Aphanizomenonaceae	Dolichospermum	sp. UHCC 0315A
seq53	144	436	249	122	84	73	0	0	0	Bacteria	Pseudomonadota	Gammaproteobacteria	Vibrionales	Vibrionaceae	Vibrio	vulnificus
seq54	75	192	158	203	190	181	27	21	41	Bacteria	Pseudomonadota	Gammaproteobacteria	Oceanospirillales	Halomonadaceae	Chromohalobacter	salexigens
seq55	105	212	155	167	193	142	34	23	29	Bacteria	Pseudomonadota	Gammaproteobacteria	Oceanospirillales	Halomonadaceae	Halomonas	sp. GT
seq56	62	301	125	153	58	93	133	20	94	Bacteria	Bacillota	Bacilli	Lactobacillales	Lactobacillaceae	Pediococcus	claussenii
seq57	100	257	153	177	200	135	15	0	0	Bacteria	Bacillota	Bacilli	Bacillales	Bacillaceae	Paraliobacillus	quinghaiensis
seq58	118	241	198	122	153	110	28	11	22	Bacteria	Pseudomonadota	Gammaproteobacteria	Thiotrichales	Piscirickettsiaceae	Methylophaga	sulfidovorans
seq59	89	516	142	63	31	42	23	0	16	Bacteria	Bacillota	Bacilli	Lactobacillales	Lactobacillaceae	Limosilactobacillus	fermentum
seq60	57	193	109	99	102	96	81	58	98	Bacteria	Bacillota	Bacilli	Lactobacillales	Lactobacillaceae	Lactobacillus	wasatchensis
seq61	53	131	97	93	128	105	74	73	111	Bacteria	Bacillota	Bacilli	Bacillales	Thermoactinomycetaceae	Laceyella	sacchari
seq62	42	51	83	112	221	233	51	17	27	Bacteria	Pseudomonadota	Alphaproteobacteria	Rickettsiales	Rickettsiaceae	Rickettsia	japonica
seq63	98	682	29	0	0	0	0	0	0	Bacteria	Bacillota	Bacilli	Lactobacillales	Carnobacteriaceae	Marinilactibacillus	piezotolerans
seq64	46	101	83	129	174	138	41	48	34	Bacteria	Pseudomonadota	Alphaproteobacteria	Rhizobiales	Aurantimonadaceae	Martelella	suaedae
seq65	82	369	158	97	40	42	0	0	0	Bacteria	Pseudomonadota	Gammaproteobacteria	Vibrionales	Vibrionaceae	Vibrio	fluvialis
seq66	61	362	198	36	16	31	41	0	38	Bacteria	Bacillota	Bacilli	Lactobacillales	Lactobacillaceae	Lactobacillus	sp. CBA3605
seq67	77	209	119	88	68	71	21	0	20	Bacteria	Pseudomonadota	Gammaproteobacteria	Oceanospirillales	Halomonadaceae	Halomonas	sulfidaeris
seq68	59	318	113	73	30	48	0	0	0	Bacteria	Pseudomonadota	Gammaproteobacteria	Vibrionales	Vibrionaceae	Catenococcus	thiocycli
seq69	67	253	101	65	57	61	0	0	0	Bacteria	Pseudomonadota	Gammaproteobacteria	Alteromonadales	Shewanellaceae	Shewanella	japonica
seq70	54	118	94	78	96	49	36	31	43	Bacteria	Bacillota	Bacilli	Lactobacillales	Lactobacillaceae	Lactobacillus	acidipiscis

seq71	59	104	88	88	86	90	19	14	25	Bacteria	Pseudomonadota	Gammaproteobacteria			Gallaecimonas	mangrovi
seq72	53	111	107	64	69	59	25	22	36	Bacteria	Pseudomonadota	Gammaproteobacteria	Oceanospirillales	Halomonadaceae	Halomonas	alkaliantarctica
seq73	73	127	104	76	88	70	0	0	0	Bacteria	Pseudomonadota	Gammaproteobacteria	Enterobacterales	Pectobacteriaceae	Sodalis	praecaptivus
seq74	26	82	87	107	80	128	24	0	0	Bacteria	Cyanobacteriota		Chroococcales	Aphanothecaceae	Euhalothece	natronophila
seq75	37	94	77	77	102	73	21	23	26	Bacteria	Pseudomonadota	Alphaproteobacteria	Rhodospirillales	Acetobacteraceae	Gluconobacter	oxydans
seq76	0	96	41	66	126	130	34	15	15	Bacteria	Cyanobacteriota		Nostocales	Nostocaceae	Anabaena	sp. WA102
seq77	14	0	35	90	207	152	0	0	0	Bacteria	Pseudomonadota	Alphaproteobacteria	Rickettsiales	Rickettsiaceae	Rickettsia	asiatica
seq78	31	146	79	70	106	63	0	0	0	Bacteria	Bacillota	Bacilli	Lactobacillales	Carnobacteriaceae	Alkalibacterium	olivapovliticus
seq79	80	168	154	22	35	30	0	0	0	Bacteria	Pseudomonadota	Gammaproteobacteria	Pseudomonadales	Pseudomonadaceae	Pseudomonas	helleri
seq80	55	121	94	65	67	41	10	10	13	Bacteria	Pseudomonadota	Gammaproteobacteria	Alteromonadales	Shewanellaceae	Shewanella	baltica
seq81	27	54	73	96	63	133	25	0	0	Bacteria	Cyanobacteriota		Nostocales	Calotrichaceae	Calothrix	sp. 336/3
seq82	38	100	62	44	61	45	53	24	44	Bacteria	Bacillota	Bacilli	Lactobacillales	Lactobacillaceae	Lactobacillus	namurensis
seq83	45	74	56	53	105	84	12	16	17	Bacteria	Bacillota	Bacilli	Lactobacillales	Streptococcaceae	Lactococcus	lactis
seq84	46	129	93	54	63	57	0	0	13	Bacteria	Pseudomonadota	Gammaproteobacteria	Oceanospirillales	Halomonadaceae	Halomonas	aquamarina
seq85	41	152	62	85	44	41	0	0	0	Bacteria	Pseudomonadota	Gammaproteobacteria	Alteromonadales	Alteromonadaceae	Marinobacter	xestospongiae
seq86	53	169	72	28	37	21	15	0	15	Bacteria	Pseudomonadota	Gammaproteobacteria	Alteromonadales	Shewanellaceae	Shewanella	dokdonensis
seq87	53	66	65	86	72	36	14	0	12	Bacteria	Pseudomonadota	Gammaproteobacteria	Enterobacterales	Erwiniaceae	Pantoea	agglomerans
seq88	19	32	45	47	84	95	36	0	17	Bacteria	Cyanobacteriota		Nostocales	Nostocaceae	Anabaena	cylindrica
seq89	30	50	66	62	64	39	14	20	26	Bacteria	Pseudomonadota	Alphaproteobacteria	Rhizobiales	Aurantimonadaceae	Martelella	mediterranea
seq90	47	105	102	36	34	38	0	0	0	Bacteria	Pseudomonadota	Gammaproteobacteria	Enterobacterales	Erwiniaceae	Pantoea	dispersa
seq91	34	133	53	33	29	28	46	0	0	Bacteria	Cyanobacteriota		Oscillatoriales	Microcoleaceae	Tychonema	bourrellyi
seq92	39	83	82	54	35	44	0	0	0	Bacteria	Pseudomonadota	Gammaproteobacteria	Alteromonadales	Idiomarinaceae	Idiomarina	xiamenensis
seq93	24	72	46	55	54	27	19	16	15	Bacteria	Bacillota	Bacilli	Lactobacillales	Leuconostocaceae	Leuconostoc	pseudomesenteroides
seq94	38	20	46	59	71	55	0	19	19	Bacteria	Actinomycetota	Actinobacteria	Micrococcales	Microbacteriaceae	Curtobacterium	sp. BH-2-1-1
seq95	31	174	34	30	18	20	0	0	0	Bacteria	Pseudomonadota	Gammaproteobacteria	Alteromonadales	Colwelliaceae	Colwellia	sp. PAMC 20917
seq96	32	146	52	44	16	16	0	0	0	Bacteria	Pseudomonadota	Gammaproteobacteria	Aeromonadales	Aeromonadaceae	Aeromonas	hydrophila
seq97	43	83	49	48	54	28	0	0	0	Bacteria	Pseudomonadota	Gammaproteobacteria	Enterobacterales	Enterobacteriaceae	Enterobacter	cloacae
seq98	22	119	49	40	16	27	17	0	10	Bacteria	Bacillota	Bacilli	Lactobacillales	Lactobacillaceae	Lactobacillus	odoratitofui
seq99	30	49	55	38	59	50	15	0	0	Bacteria	Pseudomonadota	Alphaproteobacteria	Rhodospirillales	Rhodospirillaceae	Thalassospira	lohafexi
seq100	29	129	56	36	20	22	0	0	0	Bacteria	Pseudomonadota	Gammaproteobacteria	Aeromonadales	Aeromonadaceae	Zobellella	denitrificans
seq101	21	111	70	17	17	19	15	0	19	Bacteria	Bacillota	Bacilli	Lactobacillales	Lactobacillaceae	Lactobacillus	brevis
seq102	32	94	63	28	36	35	0	0	0	Bacteria	Pseudomonadota	Gammaproteobacteria	Enterobacterales	Enterobacteriaceae	Enterobacter	asburiae
seq103	17	90	63	29	43	30	0	0	12	Bacteria	Pseudomonadota	Gammaproteobacteria			Gallaecimonas	xiamenensis
seq104	30	98	52	47	23	33	0	0	0	Bacteria	Pseudomonadota	Gammaproteobacteria	Alteromonadales	Shewanellaceae	Shewanella	pealeana
seq105	14	27	45	55	47	78	16	0	0	Bacteria	Cyanobacteriota		Nostocales			Nostocales cyanobacterium HT-58-2
seq106	41	83	63	47	16	19	0	0	0	Bacteria	Pseudomonadota	Gammaproteobacteria	Oceanospirillales	Halomonadaceae	Halomonas	venusta
seq107	0	19	45	73	67	63	0	0	0	Bacteria	Pseudomonadota	Gammaproteobacteria	Alteromonadales	Shewanellaceae	Shewanella	fodinae
seq108	26	83	52	38	32	34	0	0	0	Bacteria	Pseudomonadota	Gammaproteobacteria	Oceanospirillales	Halomonadaceae	Halomonas	titanicae
seq109	46	35	49	37	28	30	0	0	24	Bacteria	Bacillota	Bacilli	Lactobacillales	Enterococcaceae	Enterococcus	sp. FDAARGOS 375
seq110	36	36	39	53	37	35	0	0	0	Bacteria	Pseudomonadota	Gammaproteobacteria	Enterobacterales	Yersiniaceae	Rahnella	aquatilis
seq111	16	31	38	43	38	51	16	0	0	Bacteria	Cyanobacteriota		Nostocales	Nostocaceae	Cronbergia	siamensis
seq112	0	36	40	42	25	34	23	14	17	Bacteria	Bacillota	Bacilli	Lactobacillales	Lactobacillaceae	Lactobacillus	hilgardii
seq113	15	167	11	0	27	0	0	0	0	Bacteria	Bacillota	Bacilli	Lactobacillales	Leuconostocaceae	Leuconostoc	suionicum
seq114	25	68	49	38	16	23	0	0	0	Bacteria	Pseudomonadota	Gammaproteobacteria	Alteromonadales	Shewanellaceae	Shewanella	sp. SNU WT4
seq115	29	71	58	27	0	16	0	0	0	Bacteria	Pseudomonadota	Gammaproteobacteria	Oceanospirillales	Saccharospirillaceae	Saccharospirillum	mangrovi
seq116	15	81	34	0	0	12	27	0	28	Bacteria	Bacillota	Bacilli	Lactobacillales	Lactobacillaceae	Lactobacillus	oryzae
seq117	0	30	19	26	46	62	13	0	0	Bacteria	Cyanobacteriota		Nostocales	Nostocaceae	Cylindrospermum	stagnale
seq118	0	96	37	21	17	13	0	0	0	Bacteria	Pseudomonadota	Gammaproteobacteria	Vibrionales	Vibrionaceae	Vibrio	nigripulchritudo
seq119	31	41	34	28	24	23	0	0	0	Bacteria	Pseudomonadota	Gammaproteobacteria	Enterobacterales	Yersiniaceae	Ewingella	americana
seq120	0	20	12	26	20	16	43	0	41	Bacteria	Bacillota	Bacilli	Lactobacillales	Lactobacillaceae	Lactobacillus	curieae
seq121	0	53	22	37	12	50	0	0	0	Bacteria	Bacillota	Bacilli	Lactobacillales	Enterococcaceae	Enterococcus	gallinarum
seq122	30	45	30	27	20	19	0	0	0	Bacteria	Pseudomonadota	Gammaproteobacteria	Vibrionales	Vibrionaceae	Salinivibrio	sp. YCSC6
seq123	19	72	44	22	0	0	13	0	0	Bacteria	Bacillota	Bacilli	Lactobacillales	Lactobacillaceae	Pediococcus	acidilactici
seq124	17	102	32	16	0	0	0	0	0	Bacteria	Pseudomonadota	Gammaproteobacteria	Vibrionales	Vibrionaceae	Vibrio	parahaemolyticus
seq125	36	42	44	25	19	0	0	0	0	Bacteria	Pseudomonadota	Gammaproteobacteria	Alteromonadales	Alteromonadaceae	Alishewanella	longhuensis
seq126	20	48	19	14	34	26	0	0	0	Bacteria	Pseudomonadota	Gammaproteobacteria	Thiotrichales	Francisellaceae	Francisella	salina
seq127	23	107	23	0	0	0	0	0	0	Bacteria	Pseudomonadota	Gammaproteobacteria	Alteromonadales	Shewanellaceae	Shewanella	marina
seq128	13	105	19	15	0	0	0	0	0	Bacteria	Pseudomonadota	Gammaproteobacteria	Vibrionales	Vibrionaceae	Vibrio	scophthalmi
seq129	15	74	41	14	0	0	0	0	0	Bacteria	Pseudomonadota	Gammaproteobacteria	Alteromonadales	Idiomarinaceae	Idiomarina	planktonica
seq130	0	22	29	35	32	25	0	0	0	Bacteria	Pseudomonadota	Gammaproteobacteria	Enterobacterales	Enterobacteriaceae	Klebsiella	oxytoca
seq131	17	40	21	29	16	19	0	0	0	Bacteria	Pseudomonadota	Gammaproteobacteria	Alteromonadales	Ferrimonadaceae	Paraferrimonas	haliotis
seq132	26	61	39	0	15	0	0	0	0	Bacteria	Bacillota	Bacilli	Lactobacillales	Enterococcaceae	Enterococcus	saccharolyticus
seq133	26	56	22	17	17	0	0	0	0	Bacteria	Pseudomonadota	Gammaproteobacteria	Alteromonadales	Ferrimonadaceae	Ferrimonas	balearica
seq134	23	48	32	19	0	15	0	0	0	Bacteria	Bacillota	Bacilli	Lactobacillales	Lactobacillaceae	Lactobacillus	fabifermentans
seq135	0	69	26	22	15	0	0	0	0	Bacteria	Pseudomonadota	Gammaproteobacteria	Pseudomonadales	Pseudomonadaceae	Pseudomonas	putida
seq136	15	56	28	30	0	0	0	0	0	Bacteria	Pseudomonadota	Gammaproteobacteria	Alteromonadales	Shewanellaceae	Shewanella	khirikhana
seq137	24	45	23	23	0	13	0	0	0	Bacteria	Pseudomonadota	Gammaproteobacteria	Enterobacterales	Enterobacteriaceae	Enterobacter	hormaechei
seq138	27	41	29	18	0	11	0	0	0	Bacteria	Pseudomonadota	Gammaproteobacteria	Oceanospirillales	Halomonadaceae	Halomonas	hydrothermalis
seq139	17	66	32	0	0	10	0	0	0	Bacteria	Bacillota	Bacilli	Lactobacillales	Lactobacillaceae	Lactobacillus	nenjiangensis
seq140	13	28	24	23	14	16	0	0	0	Bacteria	Pseudomonadota	Gammaproteobacteria	Oceanospirillales	Halomonadaceae	Halomonas	nanhaiensis
seq141	0	40	23	18	19	14	0	0	0	Bacteria	Bacillota	Bacilli	Lactobacillales	Lactobacillaceae	Lactobacillus	zymae
seq142	12	58	22	22	0	0	0	0	0	Bacteria	Pseudomonadota	Gammaproteobacteria	Oceanospirillales	Halomonadaceae	Chromohalobacter	israelensis
seq143	18	27	24	13	17	13	0	0	0	Bacteria	Bacillota	Bacilli	Bacillales	Bacillaceae	Aquibacillus	albus

seq144	14	53	15	15	0	0	13	0	0	Bacteria	Bacillota	Bacilli	Lactobacillales	Lactobacillaceae	Lactobacillus	oligofermentans
seq145	0	23	22	21	24	20	0	0	0	Bacteria	Pseudomonadota	Alphaproteobacteria	Rhizobiales	Aurantimonadaceae	Martelella	endophytica
seq146	12	52	19	16	0	11	0	0	0	Bacteria	Pseudomonadota	Gammaproteobacteria			Gallaecimonas	pentaromativorans
seq147	0	62	30	0	0	18	0	0	0	Bacteria	Pseudomonadota	Gammaproteobacteria	Alteromonadales	Alteromonadaceae	Marinobacter	salarius
seq148	0	44	22	0	29	14	0	0	0	Bacteria	Pseudomonadota	Gammaproteobacteria	Vibrionales	Vibrionaceae	Photobacterium	damselae
seq149	15	0	26	17	26	24	0	0	0	Bacteria	Pseudomonadota	Gammaproteobacteria	Enterobacterales	Erwiniaceae	Erwinia	sp. QL-Z3
seq150	17	37	30	12	11	0	0	0	0	Bacteria	Pseudomonadota	Gammaproteobacteria	Oceanospirillales	Halomonadaceae	Salinicola	peritrichatus
seq151	12	42	24	17	12	0	0	0	0	Bacteria	Pseudomonadota	Gammaproteobacteria	Vibrionales	Vibrionaceae	Vibrio	coralliilyticus
seq152	0	83	17	0	0	0	0	0	0	Bacteria	Pseudomonadota	Gammaproteobacteria	Oceanospirillales	Halomonadaceae	Salinicola	acroporae
seq153	0	0	0	27	34	36	0	0	0	Bacteria	Cyanobacteriota		Synechococcales	Leptolyngbyaceae	Leptolyngbya	sp. O-77
seq154	0	0	0	22	29	43	0	0	0	Bacteria	Cyanobacteriota		Oscillatoriales	Cyanothecaceae	Cyanothece	sp. PCC 7425
seq155	0	16	14	27	19	15	0	0	0	Bacteria	Bacteroidota		Bacteroidetes Order II. Incertae	Rhodothermaceae	Salinibacter	ruber
seq156	0	13	0	0	40	38	0	0	0	Bacteria	Cyanobacteriota		Synechococcales	Synechococcaceae	Synechococcus	sp. SynAce01
seq157	17	47	26	0	0	0	0	0	0	Bacteria	Pseudomonadota	Gammaproteobacteria	Chromatiales		Thiohalobacter	thiocyanaticus
seq158	14	33	25	18	0	0	0	0	0	Bacteria	Pseudomonadota	Gammaproteobacteria	Enterobacterales	Erwiniaceae	Erwinia	billingiae
seq159	16	46	27	0	0	0	0	0	0	Bacteria	Pseudomonadota	Gammaproteobacteria	Enterobacterales	Enterobacteriaceae	Klebsiella	pneumoniae
seq160	0	0	13	20	25	30	0	0	0	Bacteria	Cyanobacteriota		Synechococcales	Pseudanabaenaceae	Pseudanabaena	sp. PCC 7367
seq161	0	68	20	0	0	0	0	0	0	Bacteria	Pseudomonadota	Gammaproteobacteria	Alteromonadales	Shewanellaceae	Shewanella	colwelliana
seq162	13	47	28	0	0	0	0	0	0	Bacteria	Pseudomonadota	Gammaproteobacteria	Alteromonadales	Shewanellaceae	Shewanella	halifaxensis
seq163	0	15	18	15	22	15	0	0	0	Bacteria	Actinomycetota	Actinobacteria	Micrococcales	Microbacteriaceae	Curtobacterium	pusillum
seq164	0	50	32	0	0	0	0	0	0	Bacteria	Pseudomonadota	Gammaproteobacteria	Pseudomonadales	Pseudomonadaceae	Pseudomonas	syringae
seq165	12	58	11	0	0	0	0	0	0	Bacteria	Bacillota	Bacilli	Lactobacillales	Carnobacteriaceae	Marinilactibacillus	psychrotolerans
seq166	21	24	35	0	0	0	0	0	0	Bacteria	Pseudomonadota	Gammaproteobacteria	Pseudomonadales	Pseudomonadaceae	Pseudomonas	fragi
seq167	0	27	27	0	23	0	0	0	0	Bacteria	Pseudomonadota	Gammaproteobacteria	Enterobacterales	Enterobacteriaceae	Klebsiella	aerogenes
seq168	18	16	30	12	0	0	0	0	0	Bacteria	Bacteroidota	Flavobacteriia	Flavobacteriales	Flavobacteriaceae	Myroides	sp. ZB35
seq169	0	0	17	22	11	26	0	0	0	Bacteria	Cyanobacteriota		Nostocales	Scytonemataceae	Brasilonema	bromeliae
seq170	0	76	0	0	0	0	0	0	0	Bacteria	Pseudomonadota	Gammaproteobacteria	Alteromonadales	Colwelliaceae	Colwellia	hornerae
seq171	0	18	11	16	16	14	0	0	0	Bacteria	Bacillota	Bacilli	Lactobacillales	Lactobacillaceae	Lactobacillus	paraplantarum
seq172	0	0	14	16	19	13	0	0	12	Bacteria	Pseudomonadota	Alphaproteobacteria	Rhodospirillales	Acetobacteraceae	Acetobacter	aceti
seq173	0	21	22	14	0	17	0	0	0	Bacteria	Pseudomonadota	Gammaproteobacteria	Enterobacterales	Erwiniaceae	Pantoea	sp. SO10
seq174	16	34	21	0	0	0	0	0	0	Bacteria	Pseudomonadota	Gammaproteobacteria	Alteromonadales	Alteromonadaceae	Alteromonas	australica
seq175	18	17	14	0	21	0	0	0	0	Bacteria	Pseudomonadota	Gammaproteobacteria	Xanthomonadales	Xanthomonadaceae	Stenotrophomonas	maltophilia
seq176	17	19	17	0	0	16	0	0	0	Bacteria	Pseudomonadota	Gammaproteobacteria	Enterobacterales	Erwiniaceae	Pantoea	vagans
seq177	17	28	23	0	0	0	0	0	0	Bacteria	Bacillota	Bacilli	Lactobacillales	Enterococcaceae	Enterococcus	faecium
seq178	0	19	18	0	18	13	0	0	0	Bacteria	Bacillota	Bacilli	Lactobacillales	Streptococcaceae	Lactococcus	garvieae
seq179	15	38	15	0	0	0	0	0	0	Bacteria	Pseudomonadota	Gammaproteobacteria	Enterobacterales	Yersiniaceae	Yersinia	ruckeri
seq180	0	36	17	14	0	0	0	0	0	Bacteria	Pseudomonadota	Gammaproteobacteria	Oceanospirillales	Oceanospirillaceae	Nitrincola	lacisaponensis
seq181	0	37	28	0	0	0	0	0	0	Bacteria	Pseudomonadota	Gammaproteobacteria	Thiotrichales	Piscirickettsiaceae	Methylophaga	marina
seq182	16	34	13	0	0	0	0	0	0	Bacteria	Pseudomonadota	Alphaproteobacteria	Rhodospirillales	Acetobacteraceae	Gluconobacter	frateurii
seq183	0	51	0	12	0	0	0	0	0	Bacteria	Pseudomonadota	Gammaproteobacteria	Alteromonadales	Shewanellaceae	Shewanella	carassii
seq184	0	26	0	17	16	0	0	0	0	Bacteria	Bacteroidota	Flavobacteriia	Flavobacteriales	Flavobacteriaceae	Zunongwangia	mangrovi
seq185	0	41	18	0	0	0	0	0	0	Bacteria	Pseudomonadota	Gammaproteobacteria	Oceanospirillales	Hahellaceae	Hahella	sp. KA22
seq186	0	20	12	15	0	12	0	0	0	Bacteria	Pseudomonadota	Gammaproteobacteria	Oceanospirillales	Halomonadaceae	Halomonas	urumqiensis
seq187	0	58	0	0	0	0	0	0	0	Bacteria	Pseudomonadota	Gammaproteobacteria	Pseudomonadales	Pseudomonadaceae	Pseudomonas	fluorescens
seq188	0	43	14	0	0	0	0	0	0	Bacteria	Actinomycetota	Actinobacteria	Micrococcales	Microbacteriaceae	Curtobacterium	flaccumfaciens
seq189	0	0	17	0	21	0	19	0	0	Bacteria	Bacillota	Bacilli	Lactobacillales	Enterococcaceae	Enterococcus	sp. CR-Ec1
seq190	0	33	11	0	13	0	0	0	0	Bacteria	Pseudomonadota	Gammaproteobacteria	Aeromonadales	Aeromonadaceae	Oceanimonas	sp. GK1
seq191	0	39	0	0	0	18	0	0	0	Bacteria	Pseudomonadota	Gammaproteobacteria	Alteromonadales	Pseudoalteromonadaceae	Pseudoalteromonas	tunicata
seq192	0	30	27	0	0	0	0	0	0	Bacteria	Pseudomonadota	Gammaproteobacteria	Enterobacterales	Yersiniaceae	Rahnella	sp. ERMR1:05
seq193	12	18	12	13	0	0	0	0	0	Bacteria	Pseudomonadota	Gammaproteobacteria	Oceanospirillales	Halomonadaceae	Halomonas	elongata
seq194	0	54	0	0	0	0	0	0	0	Bacteria	Bacillota	Bacilli	Lactobacillales	Lactobacillaceae	Lactobacillus	curvatus
seq195	0	0	0	16	0	10	0	12	16	Bacteria	Pseudomonadota	Alphaproteobacteria	Sphingomonadales	Sphingomonadaceae	Sphingomonas	paucimobilis
seq196	21	32	0	0	0	0	0	0	0	Bacteria	Pseudomonadota	Gammaproteobacteria	Enterobacterales	Enterobacteriaceae	Klebsiella	michiganensis
seq197	0	21	0	15	17	0	0	0	0	Bacteria	Pseudomonadota	Gammaproteobacteria	Enterobacterales	Erwiniaceae	Erwinia	persicina
seq198	0	36	16	0	0	0	0	0	0	Bacteria	Pseudomonadota	Gammaproteobacteria	Oceanospirillales	Alcanivoracaceae	Alcanivorax	dieselolei
seq199	13	16	21	0	0	0	0	0	0	Bacteria	Pseudomonadota	Gammaproteobacteria	Oceanospirillales	Halomonadaceae	Halomonas	olivaria
seq200	0	0	22	27	0	0	0	0	0	Bacteria	Pseudomonadota	Alphaproteobacteria	Rhodospirillales	Acetobacteraceae	Gluconobacter	thailandicus
seq201	0	26	22	0	0	0	0	0	0	Bacteria	Pseudomonadota	Gammaproteobacteria	Oceanospirillales	Oceanospirillaceae	Marinobacterium	sediminicola
seq202	0	0	12	21	0	14	0	0	0	Bacteria	Pseudomonadota	Gammaproteobacteria	Cardiobacteriales	Cardiobacteriaceae	Dichelobacter	nodosus
seq203	0	36	0	0	0	0	0	10	0	Bacteria	Actinomycetota	Actinobacteria	Propionibacteriales	Propionibacteriaceae	Cutibacterium	acnes
seq204	0	29	17	0	0	0	0	0	0	Bacteria	Pseudomonadota	Gammaproteobacteria	Alteromonadales	Shewanellaceae	Parashewanella	spongiae
seq205	0	27	19	0	0	0	0	0	0	Bacteria	Pseudomonadota	Gammaproteobacteria	Oceanospirillales	Halomonadaceae	Halomonas	sp. KO116
seq206	0	0	0	0	22	23	0	0	0	Bacteria	Cyanobacteriota		Oscillatoriales	Coleofasciculaceae	Geitlerinema	sp. PCC 7407
seq207	0	31	14	0	0	0	0	0	0	Bacteria	Pseudomonadota	Gammaproteobacteria	Alteromonadales	Idiomarinaceae	Idiomarina	sp. X4
seq208	0	27	18	0	0	0	0	0	0	Bacteria	Pseudomonadota	Gammaproteobacteria	Thiotrichales	Piscirickettsiaceae	Methylophaga	thalassica
seq209	0	27	0	17	0	0	0	0	0	Bacteria	Pseudomonadota	Gammaproteobacteria	Oceanospirillales	Halomonadaceae	Chromohalobacter	japonicus
seq210	19	24	0	0	0	0	0	0	0	Bacteria	Pseudomonadota	Gammaproteobacteria	Alteromonadales	Shewanellaceae	Shewanella	fidelis
seq211	0	22	0	20	0	0	0	0	0	Bacteria	Cyanobacteriota		Chroococcales	Microcystaceae	Microcystis	viridis
seq212	0	26	16	0	0	0	0	0	0	Bacteria	Pseudomonadota	Gammaproteobacteria	Oceanospirillales	Halomonadaceae	Halomonas	sp. GFAJ-1
seq213	0	13	0	0	16	13	0	0	0	Bacteria	Pseudomonadota	Gammaproteobacteria	Pseudomonadales	Pseudomonadaceae	Pseudomonas	salina
seq214	0	27	14	0	0	0	0	0	0	Bacteria	Pseudomonadota	Alphaproteobacteria	Rhodospirillales	Rhodospirillaceae	Thalassospira	xiamenensis
seq215	0	29	12	0	0	0	0	0	0	Bacteria	Pseudomonadota	Gammaproteobacteria	Alteromonadales	Shewanellaceae	Shewanella	gelidii
seq216	0	28	0	13	0	0	0	0	0	Bacteria	Pseudomonadota	Gammaproteobacteria	Cardiobacteriales	Cardiobacteriaceae	Cardiobacterium	hominis

seq217	0	17	23	0	0	0	0	0	0	Bacteria	Pseudomonadota	Betaproteobacteria	Burkholderiales	Oxalobacteraceae	Janthinobacterium	sp. LM6
seq218	0	25	0	0	15	0	0	0	0	Bacteria	Pseudomonadota	Gammaproteobacteria	Enterobacterales	Morganellaceae	Proteus	sp. CD3
seq219	0	38	0	0	0	0	0	0	0	Bacteria	Bacteroidota	Flavobacteriia	Flavobacteriales	Flavobacteriaceae	Zunongwangia	endophytica
seq220	0	38	0	0	0	0	0	0	0	Bacteria	Pseudomonadota	Gammaproteobacteria	Alteromonadales	Alteromonadaceae	Alteromonas	sp. RKMC-009
seq221	0	26	12	0	0	0	0	0	0	Bacteria	Pseudomonadota	Gammaproteobacteria	Alteromonadales	Alteromonadaceae	Bowmanella	dokdonensis
seq222	0	0	0	0	15	22	0	0	0	Bacteria	Pseudomonadota	Alphaproteobacteria	Rickettsiales	Rickettsiaceae	Rickettsia	amblyommatis
seq223	0	37	0	0	0	0	0	0	0	Bacteria	Pseudomonadota	Gammaproteobacteria	Aeromonadales	Aeromonadaceae	Aeromonas	caviae
seq224	0	14	0	23	0	0	0	0	0	Bacteria	Pseudomonadota	Gammaproteobacteria	Chromatiales	Thioalkalibacteraceae	Guyparkeria	halophila
seq225	0	22	15	0	0	0	0	0	0	Bacteria	Pseudomonadota	Gammaproteobacteria	Oceanospirillales	Halomonadaceae	Salinicola	socius
seq226	0	26	11	0	0	0	0	0	0	Bacteria	Pseudomonadota	Gammaproteobacteria	Xanthomonadales	Xanthomonadaceae	Pseudoxanthomonas	spadix
seq227	0	0	0	18	0	0	18	0	0	Bacteria	Bacillota	Bacilli	Bacillales	Bacillaceae	Peribacillus	muralis
seq228	0	0	0	0	0	36	0	0	0	Bacteria	Pseudomonadota	Alphaproteobacteria	Emcibacterales	Emcibacteraceae	Emcibacter	sp. MEBiC09520
seq229	0	0	0	14	11	11	0	0	0	Bacteria	Pseudomonadota	Alphaproteobacteria	Rhodospirillales	Acetobacteraceae	Gluconobacter	albidus
seq230	0	36	0	0	0	0	0	0	0	Bacteria	Pseudomonadota	Gammaproteobacteria	Vibrionales	Vibrionaceae	Photobacterium	lutimaris
seq231	0	20	15	0	0	0	0	0	0	Bacteria	Pseudomonadota	Gammaproteobacteria	Oceanospirillales	Oceanospirillaceae	Marinomonas	arenicola
seq232	0	0	0	0	21	13	0	0	0	Bacteria	Cyanobacteriota		Synechococcales	Pseudanabaenaceae	Pseudanabaena	sp. ABRG5-3
seq233	0	18	16	0	0	0	0	0	0	Bacteria	Pseudomonadota	Gammaproteobacteria	Alteromonadales	Ferrimonadaceae	Ferrimonas	marina
seq234	0	34	0	0	0	0	0	0	0	Bacteria	Pseudomonadota	Gammaproteobacteria	Vibrionales	Vibrionaceae	Vibrio	gazogenes
seq235	0	16	16	0	0	0	0	0	0	Bacteria	Pseudomonadota	Gammaproteobacteria	Alteromonadales	Shewanellaceae	Shewanella	algae
seq236	0	32	0	0	0	0	0	0	0	Bacteria	Pseudomonadota	Gammaproteobacteria	Vibrionales	Vibrionaceae	Vibrio	cholerae
seq237	0	0	0	0	18	13	0	0	0	Bacteria	Bacillota	Bacilli	Lactobacillales	Lactobacillaceae	Lactobacillus	sp. D1501
seq238	0	0	0	15	16	0	0	0	0	Bacteria	Pseudomonadota	Alphaproteobacteria	Rhodobacterales	Rhodobacteraceae	Paenirhodobacter	enshiensis
seq239	0	0	0	0	31	0	0	0	0	Bacteria	Pseudomonadota	Gammaproteobacteria	Aeromonadales	Aeromonadaceae	Oceanisphaera	profunda
seq240	0	0	0	0	31	0	0	0	0	Bacteria	Pseudomonadota	Gammaproteobacteria	Alteromonadales	Pseudoalteromonadaceae	Pseudoalteromonas	sp. SM9913
seq241	0	19	0	11	0	0	0	0	0	Bacteria	Pseudomonadota	Gammaproteobacteria	Alteromonadales	Alteromonadaceae	Marinobacter	hydrocarbonoclasticus
seq242	0	30	0	0	0	0	0	0	0	Bacteria	Pseudomonadota	Gammaproteobacteria	Vibrionales	Vibrionaceae	Vibrio	campbellii
seq243	0	0	14	0	0	15	0	0	0	Bacteria	Actinomycetota	Actinobacteria	Bifidobacteriales	Bifidobacteriaceae	Bifidobacterium	longum
seq244	0	29	0	0	0	0	0	0	0	Bacteria	Pseudomonadota	Gammaproteobacteria	Alteromonadales	Shewanellaceae	Shewanella	profunda
seq245	0	29	0	0	0	0	0	0	0	Bacteria	Pseudomonadota	Gammaproteobacteria	Oceanospirillales	Oceanospirillaceae	Neptuniibacter	caesariensis
seq246	0	27	0	0	0	0	0	0	0	Bacteria	Pseudomonadota	Gammaproteobacteria	Enterobacterales	Enterobacteriaceae	Citrobacter	freundii
seq247	0	15	12	0	0	0	0	0	0	Bacteria	Pseudomonadota	Gammaproteobacteria	Oceanospirillales	Halomonadaceae	Salinicola	rhizosphaerae
seq248	0	15	11	0	0	0	0	0	0	Bacteria	Bacillota	Bacilli	Lactobacillales	Lactobacillaceae	Lactobacillus	hokkaidonensis
seq249	0	26	0	0	0	0	0	0	0	Bacteria	Bacillota	Bacilli	Lactobacillales	Lactobacillaceae	Lactobacillus	salivarius
seq250	0	26	0	0	0	0	0	0	0	Bacteria	Pseudomonadota	Gammaproteobacteria	Oceanospirillales	Halomonadaceae	Halomonas	chromatireducens
seq251	0	11	0	0	0	14	0	0	0	Bacteria	Pseudomonadota	Gammaproteobacteria	Aeromonadales	Aeromonadaceae	Aeromonas	veronii
seq252	0	25	0	0	0	0	0	0	0	Bacteria	Pseudomonadota	Gammaproteobacteria	Oceanospirillales	Alcanivoracaceae	Alcanivorax	hongdengensis
seq253	0	25	0	0	0	0	0	0	0	Bacteria	Pseudomonadota	Gammaproteobacteria	Vibrionales	Vibrionaceae	Photobacterium	gaetbulicola
seq254	0	0	0	0	0	13	11	0	0	Bacteria	Bacillota	Bacilli	Lactobacillales	Lactobacillaceae	Lactobacillus	parabuchneri
seq255	0	24	0	0	0	0	0	0	0	Bacteria	Pseudomonadota	Gammaproteobacteria	Cellvibrionales	Spongiibacteraceae	Spongiibacter	sp. IMCC21906
seq256	0	24	0	0	0	0	0	0	0	Bacteria	Pseudomonadota	Gammaproteobacteria	Pasteurellales	Pasteurellaceae	Haemophilus	parainfluenzae
seq257	0	23	0	0	0	0	0	0	0	Bacteria	Bacillota	Bacilli	Bacillales	Bacillaceae	Paraliobacillus	ryukyuensis
seq258	0	23	0	0	0	0	0	0	0	Bacteria	Pseudomonadota	Alphaproteobacteria	Rhizobiales	Aurantimonadaceae	Martelella	limonii
seq259	0	0	0	0	23	0	0	0	0	Bacteria	Pseudomonadota	Alphaproteobacteria	Rickettsiales	Rickettsiaceae	Rickettsia	bellii
seq260	0	23	0	0	0	0	0	0	0	Bacteria	Pseudomonadota	Gammaproteobacteria	Alteromonadales	Pseudoalteromonadaceae	Algicola	bacteriolytica
seq261	0	23	0	0	0	0	0	0	0	Bacteria	Pseudomonadota	Gammaproteobacteria	Alteromonadales	Shewanellaceae	Shewanella	woodyi
seq262	0	12	11	0	0	0	0	0	0	Bacteria	Pseudomonadota	Gammaproteobacteria	Enterobacterales	Enterobacteriaceae		Plautia stali symbiont
seq263	0	23	0	0	0	0	0	0	0	Bacteria	Pseudomonadota	Gammaproteobacteria	Oceanospirillales	Saccharospirillaceae	Gynuella	sunshinyii
seq264	0	22	0	0	0	0	0	0	0	Bacteria	Bacillota	Bacilli	Lactobacillales	Lactobacillaceae	Lactobacillus	malefermentans
seq265	0	22	0	0	0	0	0	0	0	Bacteria	Bacillota	Bacilli	Lactobacillales	Leuconostocaceae	Weissella	confusa
seq266	0	0	0	0	0	21	0	0	0	Bacteria	Cyanobacteriota		Chroococcales	Aphanothecaceae	Gloeothece	verrucosa
seq267	0	21	0	0	0	0	0	0	0	Bacteria	Pseudomonadota	Gammaproteobacteria	Alteromonadales	Pseudoalteromonadaceae	Pseudoalteromonas	luteoviolacea
seq268	0	21	0	0	0	0	0	0	0	Bacteria	Pseudomonadota	Gammaproteobacteria	Alteromonadales	Shewanellaceae	Shewanella	sp. YLB-06
seq269	0	0	0	0	21	0	0	0	0	Bacteria	Pseudomonadota	Gammaproteobacteria	Pseudomonadales	Moraxellaceae	Acinetobacter	johnsonii
seq270	0	20	0	0	0	0	0	0	0	Bacteria	Pseudomonadota	Gammaproteobacteria	Alteromonadales	Alteromonadaceae	Alishewanella	alkalitolerans
seq271	0	20	0	0	0	0	0	0	0	Bacteria	Pseudomonadota	Gammaproteobacteria	Alteromonadales	Shewanellaceae	Shewanella	pneumatophori
seq272	0	0	0	0	19	0	0	0	0	Bacteria	Bacillota	Bacilli	Bacillales	Bacillaceae	Paraliobacillus	sp. X-1125
seq273	0	19	0	0	0	0	0	0	0	Bacteria	Bacillota	Bacilli	Lactobacillales	Lactobacillaceae	Pediococcus	pentosaceus
seq274	0	19	0	0	0	0	0	0	0	Bacteria	Pseudomonadota	Gammaproteobacteria	Alteromonadales	Shewanellaceae	Shewanella	psychrophila
seq275	0	19	0	0	0	0	0	0	0	Bacteria	Pseudomonadota	Gammaproteobacteria	Enterobacterales	Yersiniaceae	Serratia	fonticola
seq276	0	19	0	0	0	0	0	0	0	Bacteria	Pseudomonadota	Gammaproteobacteria	Oceanospirillales	Halomonadaceae	Halomonas	sp. SF2003
seq277	0	18	0	0	0	0	0	0	0	Bacteria	Bacillota	Bacilli	Lactobacillales	Carnobacteriaceae	Alkalibacterium	pelagium
seq278	0	0	0	0	0	18	0	0	0	Bacteria	Pseudomonadota	Gammaproteobacteria	Pseudomonadales	Pseudomonadaceae	Pseudomonas	koreensis
seq279	0	17	0	0	0	0	0	0	0	Bacteria	Bacillota	Bacilli	Bacillales	Bacillaceae		Bacillaceae bacterium TKL69
seq280	0	17	0	0	0	0	0	0	0	Bacteria	Pseudomonadota	Gammaproteobacteria	Alteromonadales	Shewanellaceae	Shewanella	gelidimarina
seq281	0	0	0	0	0	0	0	17	0	Bacteria	Pseudomonadota	Gammaproteobacteria	Alteromonadales	Shewanellaceae	Shewanella	sp. MEBiC00475
seq282	0	0	0	0	17	0	0	0	0	Bacteria	Pseudomonadota	Gammaproteobacteria	Enterobacterales	Enterobacteriaceae	Raoultella	ornithinolytica
seq283	0	0	0	0	0	0	16	0	0	Bacteria	Bacillota	Bacilli	Lactobacillales	Lactobacillaceae	Lactobacillus	sunkii
seq284	0	16	0	0	0	0	0	0	0	Bacteria	Pseudomonadota	Alphaproteobacteria	Sneathiellales	Sneathiellaceae	Sneathiella	chinensis
seq285	0	16	0	0	0	0	0	0	0	Bacteria	Pseudomonadota	Gammaproteobacteria	Aeromonadales	Aeromonadaceae	Aeromonas	schubertii
seq286	0	0	0	16	0	0	0	0	0	Bacteria	Pseudomonadota	Gammaproteobacteria	Enterobacterales	Enterobacteriaceae	Enterobacter	cancerogenus
seq287	0	16	0	0	0	0	0	0	0	Bacteria	Pseudomonadota	Gammaproteobacteria	Oceanospirillales	Halomonadaceae	Halomonas	muralis
seq288	0	16	0	0	0	0	0	0	0	Bacteria	Pseudomonadota	Gammaproteobacteria	Oceanospirillales	Oceanospirillaceae	Marinobacterium	aestuarii
seq289	0	0	0	0	0	15	0	0	0	Bacteria	Cyanobacteriota		Chroococcales	Microcystaceae	Microcystis	panniformis

seq290	0	0	0	0	0	15	0	0	0	Bacteria	Cyanobacteriota		Chroococcidiopsidales	Chroococcidiopsidaceae	Aliterella	antarctica
seq291	0	0	0	0	15	0	0	0	0	Bacteria	Bacillota	Bacilli	Bacillales	Bacillaceae	Peribacillus	butanolivorans
seq292	0	15	0	0	0	0	0	0	0	Bacteria	Pseudomonadota	Gammaproteobacteria	Alteromonadales	Shewanellaceae	Shewanella	sediminis
seq293	0	0	0	0	15	0	0	0	0	Bacteria	Pseudomonadota	Gammaproteobacteria	Pseudomonadales	Pseudomonadaceae	Pseudomonas	savastanoi
seq294	0	15	0	0	0	0	0	0	0	Bacteria	Pseudomonadota	Gammaproteobacteria	Pseudomonadales	Pseudomonadaceae	Pseudomonas	sp. TMW 2.1634
seq295	0	15	0	0	0	0	0	0	0	Bacteria	Pseudomonadota	Gammaproteobacteria	Vibrionales	Vibrionaceae	Salinivibrio	proteolyticus
seq296	0	14	0	0	0	0	0	0	0	Bacteria	Bacillota	Bacilli	Lactobacillales	Carnobacteriaceae	Alkalibacterium	psychrotolerans
seq297	0	0	14	0	0	0	0	0	0	Bacteria	Bacillota	Bacilli	Lactobacillales	Lactobacillaceae	Lactobacillus	brantae
seq298	0	14	0	0	0	0	0	0	0	Bacteria	Pseudomonadota	Gammaproteobacteria	Enterobacterales	Hafniaceae	Hafnia	alvei
seq299	0	14	0	0	0	0	0	0	0	Bacteria	Pseudomonadota	Gammaproteobacteria	Oceanospirillales	Oceanospirillaceae	Neptunomonas	concharum
seq300	0	0	13	0	0	0	0	0	0	Bacteria	Pseudomonadota	Gammaproteobacteria	Vibrionales	Vibrionaceae	Corallibacterium	pacifica
seq301	0	0	12	0	0	0	0	0	0	Bacteria	Bacillota	Bacilli	Lactobacillales	Carnobacteriaceae	Carnobacterium	inhibens
seq302	0	12	0	0	0	0	0	0	0	Bacteria	Bacillota	Bacilli	Lactobacillales	Enterococcaceae	Enterococcus	casseliflavus
seq303	0	12	0	0	0	0	0	0	0	Bacteria	Bacillota	Bacilli	Lactobacillales	Lactobacillaceae	Lactobacillus	hammesii
seq304	0	12	0	0	0	0	0	0	0	Bacteria	Pseudomonadota	Gammaproteobacteria	Alteromonadales	Alteromonadaceae	Marinobacter	daepoensis
seq305	0	12	0	0	0	0	0	0	0	Bacteria	Pseudomonadota	Gammaproteobacteria	Alteromonadales	Colwelliaceae	Colwellia	demingiae
seq306	0	12	0	0	0	0	0	0	0	Bacteria	Pseudomonadota	Gammaproteobacteria	Oceanospirillales	Halomonadaceae	Halomonas	alkaliphila
seq307	0	12	0	0	0	0	0	0	0	Bacteria	Pseudomonadota	Gammaproteobacteria	Vibrionales	Vibrionaceae	Photobacterium	alginatilyticum
seq308	0	0	0	0	0	11	0	0	0	Bacteria	Cyanobacteriota		Chroococcales	Aphanothecaceae	Gloeothece	membranacea
seq309	0	0	0	0	11	0	0	0	0	Bacteria	Bacillota	Bacilli	Lactobacillales	Lactobacillaceae	Lactobacillus	silagei
seq310	0	11	0	0	0	0	0	0	0	Bacteria	Pseudomonadota	Gammaproteobacteria	Alteromonadales	Alteromonadaceae	Alishewanella	solinquinati
seq311	0	11	0	0	0	0	0	0	0	Bacteria	Pseudomonadota	Gammaproteobacteria	Alteromonadales	Colwelliaceae	Litorilituus	lipolyticus
seq312	0	0	11	0	0	0	0	0	0	Bacteria	Pseudomonadota	Gammaproteobacteria	Alteromonadales	Idiomarinaceae	Idiomarina	aestuarii
seq313	11	0	0	0	0	0	0	0	0	Bacteria	Pseudomonadota	Gammaproteobacteria	Enterobacterales	Enterobacteriaceae	Enterobacter	ludwigii
seq314	0	10	0	0	0	0	0	0	0	Bacteria	Pseudomonadota	Gammaproteobacteria	Oceanospirillales	Halomonadaceae	Kushneria	marisflavi
seq315	0	10	0	0	0	0	0	0	0	Bacteria	Pseudomonadota	Gammaproteobacteria	Oceanospirillales	Kangiellaceae	Aliikangiella	marina

Table S4: Comparison of taxonomical assignment for core microbiome species belonging to Emu, Kraken2 and dnabarcoder taxonomic classifiers. Taxonomic identification at genus e/o species level is indicated as assigned (+) or not available (n.a.).

**Marker**	**Taxon**	**Emu**		**Kraken2**		**dnabarcoder**	
		**genus**	**species**	**genus**	**species**	**genus**	**species**
16S	*Secundilactobacillus collinoides/paracollinoides* group	+	+	+	+	n.a.	n.a.
16S	*Lactiplantibacillus plantarum* group	+	+	+	+	n.a.	n.a.
16S	*Pediococcus parvulus*	+	+	+	+	n.a.	n.a.
16S	*Pediococcus damnosus*	+	+	+	+	n.a.	n.a.
16S	*Paucilactobacillus suebicus*	+	+	+	+	n.a.	n.a.
ITS	*Pichia membranifaciens*	+	+	+	n.a.	+	+
ITS	*Wickerhamomyces anomalus*	+	+	n.a.	n.a.	+	+
ITS	*Pichia manshurica*	+	+	+	n.a.	+	+

Table S5. ASV table of fungal population

	M3ITSa	M3ITSb	M3ITSc	M4ITSa	M4ITSb	M4ITSc	M6ITSa	M6ITSb	M6ITSc	Kingdom	Phylum	Class	Order	Family	Genus	Species
seq1	182720	291766	326847	60551	33196	68315	48290	27661	82499	Fungi	Ascomycota	Saccharomycetes	Saccharomycetales	Pichiaceae	Pichia	membranifaciens
seq2	154965	193950	232020	76205	42881	86181	28564	20169	58661	Fungi	Ascomycota	Saccharomycetes	Saccharomycetales	Phaffomycetaceae	Wickerhamomyces	anomalus
seq3	28235	40508	51120	20562	11364	24416	12056	7225	22463	Fungi	Ascomycota	Saccharomycetes	Saccharomycetales	Pichiaceae	Pichia	manshurica
seq4	24093	26312	28528	12715	7909	15308	6959	4426	10563	Fungi	Ascomycota	Saccharomycetes	Saccharomycetales			
seq5	7832	12427	15999	1890	1001	2352	990	737	2160	Fungi	Ascomycota	Saccharomycetes	Saccharomycetales	Pichiaceae	Pichia	
seq6	5602	6383	7139	4565	2019	4562	2738	1769	5335	Fungi	Ascomycota	Saccharomycetes	Saccharomycetales	Pichiaceae	Dekkera	bruxellensis
seq7	1870	1898	2676	4412	1093	2102	492	409	1175	Fungi	Ascomycota	Saccharomycetes	Saccharomycetales	Saccharomycetaceae	Saccharomyces	cerevisiae
seq8	1421	1750	2535	941	367	968	228	234	791	Fungi	Ascomycota	Saccharomycetes	Saccharomycetales	Saccharomycetaceae	Torulaspora	delbrueckii
seq9	875	2082	1975	301	159	354	82	45	111	Fungi	Ascomycota	Saccharomycetes	Saccharomycetales	Saccharomycetales_fam_Incertae_sedis	Candida	thaimueangensis
seq10	174	271	825	698	369	236	1233	341	232	Fungi	Ascomycota					
seq11	718	865	831	392	207	417	174	132	403	Fungi	Ascomycota	Saccharomycetes	Saccharomycetales	Debaryomycetaceae	Meyerozyma	guilliermondii
seq12	82	175	1314	378	196	453	336	76	269	Fungi	Ascomycota	Eurotiomycetes	Eurotiales	Aspergillaceae	Penicillium	roqueforti
seq13	526	598	592	224	98	215	75	62	181	Fungi	Ascomycota	Saccharomycetes	Saccharomycetales	Debaryomycetaceae	Yamadazyma	olivae
seq14	248	233	783	288	130	241	300	97	219	Fungi	Ascomycota	Saccharomycetes	Saccharomycetales	Trichomonascaceae	Zygoascus	hellenicus
seq15	185	746	820	69	38	22	115	28	19	Fungi	Ascomycota	Saccharomycetes	Saccharomycetales	Dipodascaceae		
seq16	659	652	198	106	40	142	46	14	100	Fungi	Ascomycota	Leotiomycetes	Erysiphales	Erysiphaceae	Blumeria	
seq17	129	134	425	202	111	207	128	93	251	Fungi	Ascomycota	Saccharomycetes	Saccharomycetales	Debaryomycetaceae	Priceomyces	carsonii
seq18	194	186	217	134	80	155	73	53	115	Fungi	Ascomycota	Saccharomycetes	Saccharomycetales	Saccharomycetales_fam_Incertae_sedis	Candida	atlantica
seq19	132	149	157	49	28	68	43	21	60	Fungi	Ascomycota	Saccharomycetes	Saccharomycetales	Saccharomycetales_fam_Incertae_sedis	Candida	diddensiae
seq20	93	134	147	59	49	91	22	0	53	Fungi	Ascomycota	Saccharomycetes	Saccharomycetales	Saccharomycetaceae	Kluyveromyces	lactis
seq21	93	173	222	26	0	40	17	13	32	Fungi	Ascomycota	Saccharomycetes	Saccharomycetales	Saccharomycetaceae	Issatchenkia	orientalis
seq22	35	94	150	28	28	0	28	13	0	Fungi	Ascomycota	Saccharomycetes	Saccharomycetales	Dipodascaceae	Dipodascus	geotrichum
seq23	42	68	91	20	0	22	0	0	0	Fungi	Ascomycota	Saccharomycetes	Saccharomycetales	Debaryomycetaceae	Debaryomyces	hansenii
seq24	37	27	36	13	0	14	0	0	24	Fungi	Ascomycota	Saccharomycetes	Saccharomycetales	Debaryomycetaceae	Yamadazyma	barbieri
seq25	20	22	21	11	0	26	11	0	22	Fungi	Ascomycota	Saccharomycetes	Saccharomycetales	Saccharomycetales_fam_Incertae_sedis	Candida	taylorii
seq26	18	28	58	13	0	0	0	0	14	Fungi	Ascomycota	Sordariomycetes	Hypocreales	Nectriaceae	Fusarium	waltergamsii
seq27	34	54	16	0	0	0	0	0	0	Fungi	Ascomycota	Dothideomycetes	Capnodiales	Mycosphaerellaceae	Ramularia	plurivora
seq28	17	29	24	10	0	0	10	0	0	Fungi	Ascomycota	Saccharomycetes	Saccharomycetales	Debaryomycetaceae	Millerozyma	farinosa
seq29	18	37	14	0	0	0	0	0	16	Fungi	Ascomycota	Saccharomycetes	Saccharomycetales	Metschnikowiaceae	Kodamaea	ohmeri
seq30	15	12	25	0	0	31	0	0	0	Fungi	Ascomycota	Saccharomycetes	Saccharomycetales	Debaryomycetaceae	Schwanniomyces	etchellsii
seq31	10	14	30	0	0	0	0	0	10	Fungi	Ascomycota	Saccharomycetes	Saccharomycetales	Saccharomycopsidaceae	Saccharomycopsis	olivae
seq32	0	13	18	0	0	19	0	0	0	Fungi	Ascomycota	Saccharomycetes	Saccharomycetales	Metschnikowiaceae	Metschnikowia	pulcherrima
seq33	14	18	12	0	0	0	0	0	0	Fungi	Basidiomycota	Tremellomycetes	Tremellales	Bulleribasidiaceae	Vishniacozyma	carnescens
seq34	0	42	0	0	0	0	0	0	0	Fungi	Basidiomycota	Malasseziomycetes	Malasseziales	Malasseziaceae	Malassezia	globosa
seq35	0	20	18	0	0	0	0	0	0	Fungi	Ascomycota	Saccharomycetes	Saccharomycetales	Metschnikowiaceae	Metschnikowia	ziziphicola
seq36	0	16	21	0	0	0	0	0	0	Fungi	Ascomycota	Saccharomycetes	Saccharomycetales	Debaryomycetaceae	Meyerozyma	caribbica
seq37	0	16	17	0	0	0	0	0	0	Fungi	Ascomycota	Dothideomycetes	Dothideales	Aureobasidiaceae	Aureobasidium	pullulans
seq38	0	10	13	0	0	0	0	0	0	Fungi	Ascomycota	Saccharomycetes	Saccharomycetales	Pichiaceae	Pichia	myanmarensis
seq39	0	11	0	0	0	12	0	0	0	Fungi	Basidiomycota	Malasseziomycetes	Malasseziales	Malasseziaceae	Malassezia	restricta
seq40	0	12	10	0	0	0	0	0	0	Fungi	Ascomycota	Saccharomycetes	Saccharomycetales	Pichiaceae	Ogataea	wangdongensis
seq41	0	19	0	0	0	0	0	0	0	Fungi	Ascomycota	Saccharomycetes	Saccharomycetales	Saccharomycetaceae	Saccharomyces	mikatae
seq42	0	18	0	0	0	0	0	0	0	Fungi	Ascomycota	Saccharomycetes	Saccharomycetales	Trichomonascaceae	Wickerhamiella	
seq43	0	0	0	0	0	0	0	17	0	Fungi	Basidiomycota	Malasseziomycetes	Malasseziales	Malasseziaceae	Malassezia	arunalokei
seq44	0	0	0	0	0	14	0	0	0	Fungi	Ascomycota	Eurotiomycetes	Eurotiales	Aspergillaceae	Penicillium	kongii
seq45	0	0	13	0	0	0	0	0	0	Fungi						
seq46	0	0	0	0	0	0	13	0	0	Fungi	Ascomycota	Saccharomycetes	Saccharomycetales	Dipodascaceae	Galactomyces	
seq47	0	13	0	0	0	0	0	0	0	Fungi	Ascomycota	Saccharomycetes	Saccharomycetales	Saccharomycetales_fam_Incertae_sedis	Candida	spencermartinsiae
seq48	0	0	0	0	0	0	0	0	12	Fungi	Ascomycota	Eurotiomycetes	Eurotiales	Aspergillaceae	Aspergillus	melleus
seq49	0	0	0	0	0	12	0	0	0	Fungi	Ascomycota	Saccharomycetes	Saccharomycetales	Saccharomycetales_fam_Incertae_sedis	Candida	tropicalis
seq50	0	0	11	0	0	0	0	0	0	Fungi	Ascomycota	Saccharomycetes	Saccharomycetales	Phaffomycetaceae	Starmera	stellimalicola
seq51	0	0	0	0	0	10	0	0	0	Fungi	Ascomycota	Eurotiomycetes	Eurotiales	Aspergillaceae	Aspergillus	versicolor
seq52	0	0	0	0	0	0	0	10	0	Fungi	Basidiomycota	Malasseziomycetes	Malasseziales	Malasseziaceae		
